# Acevaltrate as a novel ferroptosis inducer with dual targets of PCBP1/2 and GPX4 in colorectal cancer

**DOI:** 10.1038/s41392-025-02296-7

**Published:** 2025-07-07

**Authors:** Dianping Yu, Hongmei Hu, Qing Zhang, Chengji Wang, Mengting Xu, Hanchen Xu, Xiangxin Geng, Minchen Cai, Hongwei Zhang, Mengmeng Guo, Dong Lu, Hanchi Xu, Linyang Li, Xing Zhang, Ruling Shen, Sheng Lin, Qun Wang, Weidong Zhang, Sanhong Liu

**Affiliations:** 1 State Key Laboratory of Discovery and Utilization of Functional Components in Traditional Chinese Medicine, Shanghai Frontiers Science Center of TCM Chemical Biology, Institute of Interdisciplinary Integrative Medicine Research, Shanghai, China; 2 Institute of Digestive Diseases, Longhua Hospital, Shanghai University of Traditional Chinese Medicine, Shanghai, China; 3 Shanghai Laboratory Animal Research Center, Shanghai, China; 4 Key Laboratory of Chinese Internal Medicine of Ministry of Education and Beijing, Dongzhimen Hospital, Beijing University of Chinese Medicine, Beijing, China; 5 Department of Phytochemistry, School of Pharmacy, Second Military Medical University, Shanghai, China; 6 Institute of Medicinal Plant Development, Chinese Academy of Medical Sciences and Peking Union Medical College, Beijing, China

**Keywords:** Gastrointestinal cancer, Gastrointestinal cancer

## Abstract

Ferroptosis induced by ferrous ions (Fe^2+^) and lipid peroxidation accumulation is a novel form of regulated cell death that has become a hot topic in tumor therapy research. Identifying small-molecule drugs that can induce ferroptosis in tumor cells is a very attractive therapeutic strategy. Here, we screened a natural product, acevaltrate (ACE), which rapidly and strongly induces ferroptosis in colorectal cancer cells. ACE not only increases Fe^2+^ levels in colorectal cancer cells by targeting iron chaperones PCBP1/2 and reducing their expression but also disrupts the antioxidant system of colorectal cancer cells by targeting GPX4 and inhibiting its enzymatic activity, leading to its ubiquitin-mediated degradation. This dual effect of ACE makes it significantly more effective than classical ferroptosis inducers in inducing ferroptosis. Our animal experiments revealed that the therapeutic effect of ACE surpasses that of established ferroptosis-inducing drugs and is superior to that of first-line clinical drugs such as capecitabine and TAS-102. Importantly, ACE also demonstrated superior inhibitory effects in colorectal tumor organoids versus at the cellular level, underscoring its potential for clinical application. This study pioneers the discovery of a small molecule inhibitor that targets both PCBP1/2 and GPX4, offering a novel therapeutic strategy for eliminating cancer cells through ferroptosis.

**Figure:**
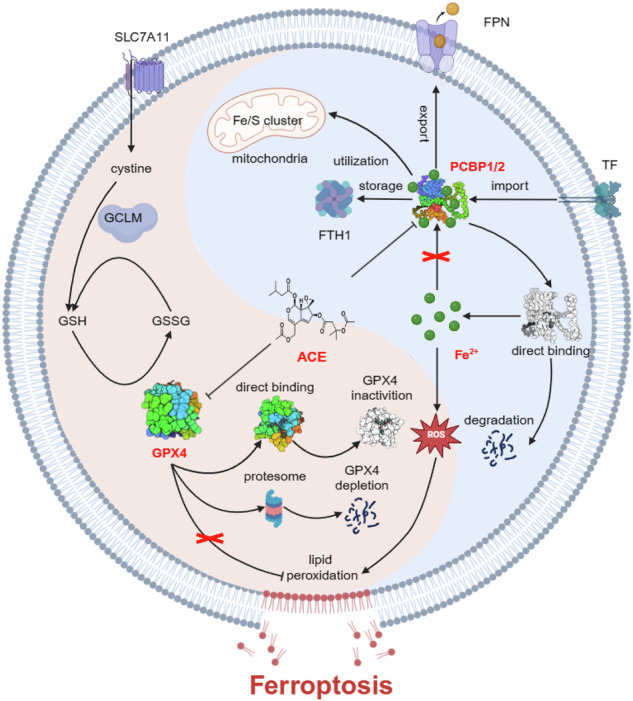
Acevaltrate (ACE) was identified as a potent inducer of ferroptosis in colorectal cancer cells. ACE increases Fe^2+^ levels by targeting PCBP1/2 and disrupts the antioxidant system by inhibiting GPX4, leading to its degradation. This dual action makes ACE more effective at inducing ferroptosis than traditional inducers. Our study introduces ACE as the first small-molecule inhibitor of PCBP1/2 and GPX4, offering a new therapeutic approach for cancer cell elimination through ferroptosis

Acevaltrate (ACE) was identified as a potent inducer of ferroptosis in colorectal cancer cells. ACE increases Fe^2+^ levels by targeting PCBP1/2 and disrupts the antioxidant system by inhibiting GPX4, leading to its degradation. This dual action makes ACE more effective at inducing ferroptosis than traditional inducers. Our study introduces ACE as the first small-molecule inhibitor of PCBP1/2 and GPX4, offering a new therapeutic approach for cancer cell elimination through ferroptosis

## Introduction

Over the past decade, ferroptosis has been widely recognized as an emerging antitumor therapeutic strategy. In contrast to caspase-3-induced apoptosis and receptor-interacting protein kinase 3 (RIPK3)-induced necroptosis, ferroptosis relies on unrestricted lipid peroxidation.^1^ High intracellular levels of ferrous ions and reactive oxygen species (ROS) are among the features that distinguish ferroptosis from other forms of cell death.^2^ They maintain not only the proliferation of tumor cells but also the lipid peroxidation of membrane phospholipids.^3,4^ Therefore, ferrous ions promote the accumulation of ROS in tumor cells through the Fenton reaction, which may have an excellent antitumor effect. Glutathione peroxidase 4 (GPX4) utilizes glutathione (GSH) to reduce lipid peroxidation to lipid alcohols, so drugs that target GPX4 or GSH to accumulate lipid peroxides offer the possibility of fighting cancer.^4,5^ Studies have shown that lipid peroxidation may act as a “find me” signal to promote antigen presentation and enhance tumor immunotherapy.^6^ In addition, epithelial-mesenchymal transition (EMT)-mediated tumor metastasis and drug resistance result in tumor cell sensitivity to ferroptosis.^5,7^ Indeed, numerous studies have shown that ferroptosis inducers such as RSL3 and erastin can induce ferroptosis in mouse tumor models and human tumor cell lines by inducing lipid peroxidation.^8,9^ In addition, ferroptosis inducers can also increase the antitumor activity of chemotherapeutic agents.^5^ Despite the significant progress that has been achieved, the effectiveness of a single GPX4 inhibition strategy is limited due to the discovery of more antioxidant systems and the regulation of amino acid metabolism, lipid metabolism, and iron metabolism.^2,10,11^ Therefore, targeting ferroptosis to suppress tumor progression remains a challenge, and the development of ferroptosis-inducing agents that target multiple pathways to improve therapeutic efficacy is crucial.

In addition to inhibiting the antioxidant system, increasing the level of Fe^2+^ in cells is another pathway for ferroptosis induction. For example, ferric ions (Fe^3+^) are transported into the cell through transferrin (TF) and then reduced to Fe^2+^ by the metal reductase six-transmembrane epithelial antigen of the prostate 3 (STEAP3). Ferrous ions (Fe^2+^) are then transported to the cytoplasm by the metal transporter protein DMT1.^2^ It has also been shown that nuclear receptor coactivator 4 (NCOA4) releases a large amount of labile iron to induce ferroptosis through lysosomal degradation of ferritin heavy chain 1 (FTH1). Moreover, heme oxygenase 1 (HO-1) generates Fe^2+^ to induce lipid peroxidation by catalyzing heme degradation. For example, BAY mediates ferroptosis in breast cancer cells by causing cellular ferrous iron accumulation via the upregulation of HO-1,^12^ and the knockdown of NCOA4 suppresses erastin-induced ferroptosis.^13^ Although the above pathways effectively increase intracellular Fe^2+^ levels, these pathways constitute a very small part of iron metabolism, which includes the uptake, export, storage, and utilization of iron.^14^ The poly(C)-binding protein (PCBP) family consists of four proteins: PCBP1, PCBP2, PCBP3, and PCBP4. PCBP1 and PCBP2, also known as iron chaperonin proteins, play important roles in the storage, utilization, and export of Fe^2+^.^15^ In addition, the regulation of iron by NCOA4 and HO-1 is also associated with PCBP.^16,17^ Disruption of PCBP may rapidly release ferrous ions, and downregulation of PCBP1/2 was shown to induce ferroptosis in malignant mesothelioma and head and neck cancer.^18,19^ Colorectal cancer (CRC) is a prevalent malignant tumor globally and is responsible for approximately 9.4% of cancer-related deaths.^20^ Although novel chemotherapeutic agents, molecularly targeted drugs, and immunotherapies have been used for tumor treatment, drug resistance, recurrence, and distant metastasis continue to pose significant challenges in the clinical management of colorectal cancer.^21^ High expression of PCBP1 and PCBP2 in cells is associated with the migration and invasion of various tumors.^22–25^ Additionally, PCBP1 and PCBP2 promote colorectal cell resistance to oxaliplatin or fluorouracil.^26,27^ In addition, tumor cells, especially colorectal cancer cells, have been found to have higher iron levels than nonmalignant cells.^28^ Therefore, targeting PCBP is expected to be an effective method for treating colorectal cancer.

In this study, we screened acevaltrate (ACE), a natural product isolated from Valeriana jatamansi, from a library of natural product compounds with promising antitumor effects. In previous studies, ACE was shown to have antitumor effects on various cancer types.^29^ In addition, acevaltrate has been shown to induce cell apoptosis and inhibit proliferation through the HIF-1α, Otub1/c-Maf, and USP10/CCND1 axes.^30,31^ Therefore, ACE is considered a promising natural antitumor compound. However, its potential antitumor mechanism remains unclear. We found that ACE not only increased Fe^2+^ levels by targeting PCBP1 and PCBP2 but also targeted GPX4 leading to its degradation by the ubiquitin-proteasome pathway. Furthermore, as the first identified small molecule inhibitors of PCBP1 and PCBP2, we evaluated the efficacy and safety of ACE-induced ferroptosis in tumor therapy. Our study may provide new perspectives for the development of novel therapies for refractory cancer cells.

## Results

### ACE inhibits proliferation and promotes cell death in colorectal cancer cells

**Fig. 1:**
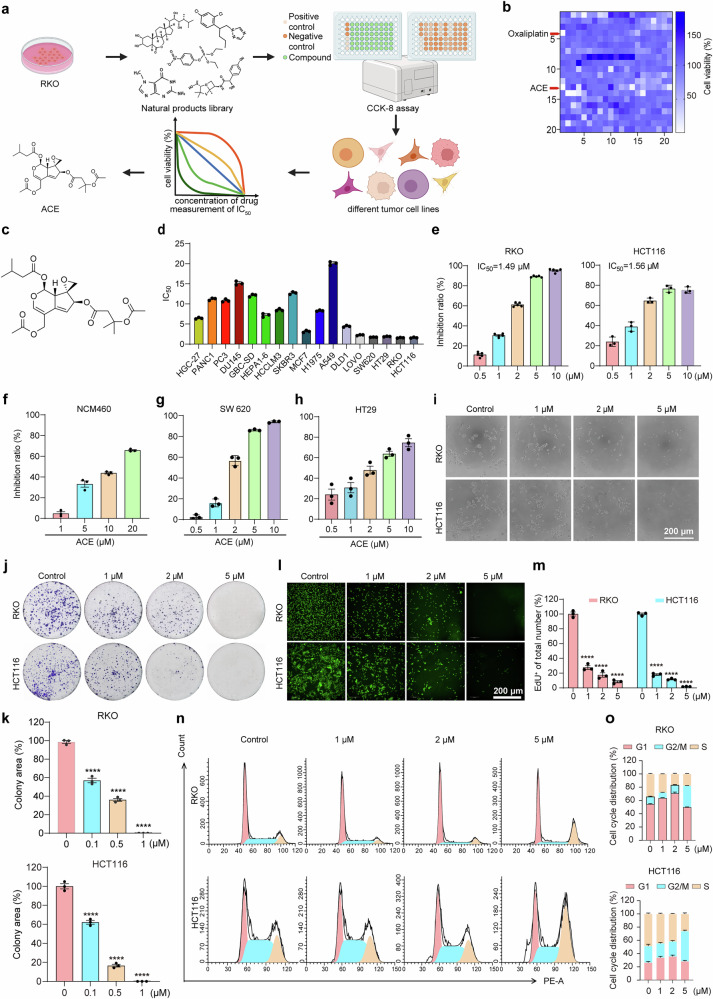
ACE inhibits cell migration and drug resistance. **a** Flow chart of the screening strategy. RKO cells were treated with a library of 420 natural compounds (10 μM) for 24 h. Cell viability was determined via a CCK-8 assay. The IC_50_ values of the candidates in different tumor cell lines were further examined. **b** Identification of antitumor compounds in the natural compound library for which the cell viability was lower than 20%. A change in color from blue to white represents a decrease in cell viability. **c** Structure of ACE. **d** IC_50_ values of ACE in different tumor cell lines. (n = 3, error bars represent SEM). **e**-**h** The inhibition ratio and IC_50_ of ACE (24 h) in HCT116 and RKO cells were measured via the CCK-8 assay. Inhibition ratios of NCM460 (**f**), SW620 (**g**), and HT29 (**h**) cells after ACE treatment for 24 h. (n = 3, error bars represent SEM). **i** Microscope images showing morphological changes in ACE-treated RKO and HCT116 cells. Scale bars, 200 μm. **j**, **k** RKO and HCT116 cells were incubated with ACE (0.1, 0.5, or 1 μM) in 12-well plates for 10 days, and the colony-forming potential of the tumor cells was assessed via crystal violet staining. (n = 3, error bars represent SEM, one-way ANOVA). **l, m** ACE (1, 2, or 5 μM) was incubated with RKO and HCT116 cells in 12-well plates for 24 h, and the proliferation of the tumor cells was detected via an EdU kit. (Scale bars, 200 μm. n = 3, error bars represent SEM, two-way ANOVA) **n**, **o** Cell cycle analysis and statistical analysis of RKO and HCT116 cells treated with ACE (1, 2, or 5 μM) for 24 h via flow cytometry. n = 3, data are shown as the mean ± SEM. The experiments consisted of three biological replicates with similar results. ****P < 0.0001

Inspired by the problems of drug resistance, metastasis, and side effects of paclitaxel, oxaliplatin, and 5-FU and the advantages of the specificity and broad spectrum of natural drugs used in cancer treatment, we selected RKO cells, a poorly differentiated human colon cancer cell line sensitive to cell death, to screen for antitumor activity in a natural compound library. We incubated RKO cells with various compounds (10 μM) for 24 h and assessed their viability via a Cell Counting Kit-8 (CCK-8) assay. As shown in Fig. 1a, b, 13 compounds were identified as candidate compounds using the criterion of an antitumor activity exceeding 80%. Furthermore, we identified ACE as the best compound with broad-spectrum antitumor effects after measuring the semi-inhibitory concentrations (IC_50_) of the compounds in different tumor cell lines (Fig. 1c, d). Unexpectedly, we found that ACE is more effective against colorectal cancer cells, with IC_50_ values ranging from 1.4 μM to 1.9 μM, and is almost nontoxic to normal intestinal epithelial cells, suggesting that ACE is more suitable for colorectal cancer treatment (Fig. 1e–i).

To determine the anti-proliferative effect of ACE, we conducted a colony formation assay and 5-ethynyl-20-deoxyuridine (EdU) assay and revealed that ACE significantly reduced colony formation efficiency and cell growth (Fig. 1j–m). In addition, flow cytometry revealed that ACE significantly induced G2/M phase arrest in HCT116 and RKO cells (Fig. 1n, o). Next, the Annexin V-FITC/PI double-staining assay revealed that ACE treatment induced cell death in HCT116 and RKO cells in a concentration-dependent manner, which was also confirmed by the calcein/PI double-staining assay (Supplementary Fig. 1a–d). In addition, ACE significantly inhibited the growth of oxaliplatin-resistant HCT116 cells (Supplementary Fig. 1e). Furthermore, ACE significantly decreased the migratory ability of CT26 and LoVo cells and downregulated the expression of EMT pathway proteins (Supplementary Fig. 1f–i). In conclusion, ACE inhibited cell proliferation, promoted cell cycle arrest, induced cell death, and inhibited cell migration and drug resistance.

### ACE induces specific ferroptosis in colorectal cancer cells

**Fig. 2:**
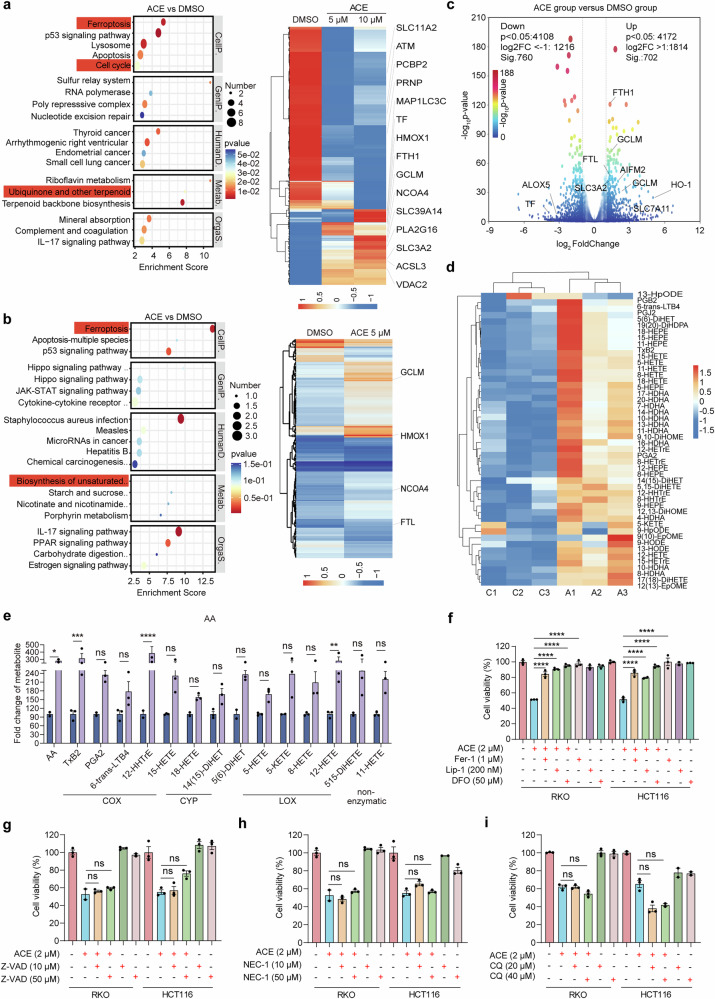
ACE induces ferroptosis in colorectal cancer cells. **a, b** Potential pathway analysis by KEGG enrichment in RKO (**a**) and HCT116 (**b**) cells and a heatmap showing differentially expressed proteins. Ferroptosis-related genes were labeled (fold change ≥1.2, unique peptides ≥2). **c** Volcano plot showing ferroptosis pathway gene expression in ACE-treated MCF7 cells (fold change ≥2, p value ≤ 0.05). **d** Heatmap showing oxidized polyunsaturated fatty acids after ACE (5 μM) treatment for 12 h. **e** AA and their metabolite levels in RKO cells treated with ACE (5 μM) for 12 h compared with DMSO. (n = 3, error bars represent SEM, Student’s t-test). **f-i** Viability of RKO and HCT116 cells treated with ferroptosis inhibitors (Fer-1: ferrostatin-1; Lip-1: liproxstatin-1; DFO: deferoxamine mesylate). (**f**) Z-VAD-FMK (**g**), NEC-1 (**h**), and CQ (**i**) after treatment with ACE (2 μM). The data are shown as the mean ± SEM. (n = 3, error bars represent SEM, one-way ANOVA). *P < 0.05, **P < 0.01, ***P < 0.001, ****P < 0.0001; ns not significant

Although ACE significantly inhibited cell proliferation and induced cell death, unlike previous reports, ACE did not activate the classical apoptotic pathway (Supplementary Fig. 1j, k). We further explored its potential non-apoptotic death mechanism. Next, we used a multiomics-based strategy (proteomics, transcriptomics, and metabolomics) to investigate the possible pathways of ACE-induced cell death. KEGG enrichment analysis of the proteomic data revealed that the ferroptosis pathway was the most enriched pathway, and the ubiquinone biosynthesis and unsaturated fatty acid biosynthesis pathways related to ferroptosis were also enriched in the ACE treatment group (Fig. 2a, b). In addition, GO enrichment analysis revealed that cellular components such as cell membranes and mitochondria were strongly associated with ferroptosis (Supplementary Fig. 2a, b). As expected, transcriptomic analysis revealed that the ferroptosis signaling pathway was significantly activated after ACE treatment (Fig. 2c and Supplementary Fig. 2c). On the basis of the important role of ferroptosis in overcoming tumor drug resistance, we tentatively hypothesized that ACE may induce ferroptosis in colorectal cancer cells.

Since lipid peroxidation is essential for ferroptosis, we next analyzed the changes in the levels of oxidized fatty acids and redox agents in RKO cells in the presence or absence of ACE. First, we observed that ACE treatment led to a significant increase in the levels of oxidized polyunsaturated fatty acids, particularly arachidonic acid (AA) and linoleic acid (LA), reflecting the accumulation of intracellular lipid peroxidation products (Fig. 2d, e and Supplementary Fig. 2d–f). Second, antioxidants were significantly elevated, suggesting that ACE-mediated lipid peroxidation may rely on increased synthesis rather than decreased scavenging (Supplementary Fig. 2g). Notably, the levels of nonenzymatic metabolites such as 9-HODE and 12-HDHA increased significantly after ACE treatment (Supplementary Fig. 2h). The multiomics results also revealed that ACE significantly regulated HO-1 and glutamate-cysteine ligase modifier subunit (GCLM) (Supplementary Fig. 2i). Furthermore, as shown in Fig. 2f–i, the cytotoxicity of ACE was blocked by ferroptosis inhibitors but not by apoptosis, necroptosis, or autophagy inhibitors. These results suggest that ACE-induced specific ferroptosis is a key determinant of colorectal cancer cell death and is largely dependent on Fe^2+^.

### ACE induces canonical and noncanonical ferroptosis

**Fig. 3:**
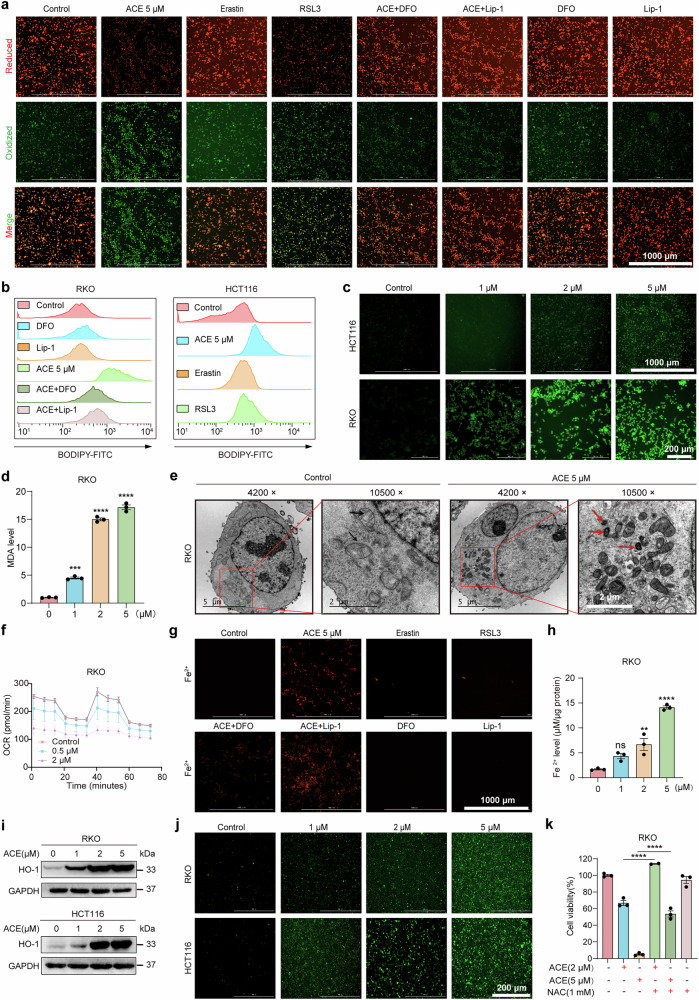
ACE induces ferroptosis by inactivating GPX4 and accumulating Fe^2+^. **a, b** BODIPY-C11 staining (**a**) and flow cytometry (**b**) showing RKO cells treated with ACE (5 μM) with or without a ferroptosis inhibitor (Lip-1, DFO) or ferroptosis inducer (RSL3, erastin) for 2 h. (n = 3, scale bars, 1000 μm). **c** Liperfluo staining for analysis of lipid peroxidation in RKO and HCT116 cells after treatment with ACE (1, 2, or 5 μM) for 2 h. (n = 3, scale bars, 1000 μm). **d** Malondialdehyde (MDA) assay showing the lipid peroxidation levels of RKO cells treated with ACE. (n = 3, error bars represent SEM, one-way ANOVA). **e** Transmission electron microscopy images of RKO cells treated with ACE (5 μM, 12 h) and DMSO (12 h). 4200×: for the observation of intact individual cell morphology. 10500×: for the observation of clear mitochondrial morphology. Red arrowheads, mitochondrial atrophy with reduced cristae; black arrowheads, normal mitochondria. Seven cells per treatment condition were examined; scale bars, 5 μm. **f** Mitochondrial respiration was measured in ACE-treated (0.5 and 2 μM, 12 h) and DMSO-treated (Ctrl) RKO cells via a Seahorse XF96 system. The cells were treated with the indicated reagents (oligo: oligomycin, ATP synthase inhibitor). FCCP, mitochondrial oxidative phosphorylation uncoupler. Rot/AA: Rotenone and antimycin A, a mitochondrial respiratory chain inhibitor, were used to measure the basal oxygen consumption rate (OCR) and maximal respiration. (n = 3, error bars represent SEM, two-way ANOVA). **g** FerroOrange staining showing RKO cells treated with ACE (5 μM) with or without a ferroptosis inhibitor (Lip-1, DFO) or ferroptosis inducer (RSL3, erastin) for 24 h (n = 3, scale bars, 1000 μm). **h** Quantitative analysis of Fe^2+^ levels in RKO cells treated with ACE via a ferrous ion colorimetric assay. (n = 3, error bars represent SEM, one-way ANOVA). **i** HO-1 protein levels in RKO and HCT116 cells after dose-dependent ACE treatment. **j** DCFH-DA staining showing the intracellular ROS levels in RKO and HCT116 cells treated with ACE (1, 2, or 5 μM) for 2 h. (n = 3, scale bars, 200 μm). **k** RKO cells were treated with ACE (2, 5 μM) with or without the ROS scavenger NAC for 24 h, and cell viability was assayed via a CCK-8 assay. (n = 3, error bars represent SEM, one-way ANOVA). **P < 0.01, ***P < 0.001, ****P < 0.0001; ns not significant

On the basis of the significant enrichment of the ferroptosis pathway, we verified whether ACE induced ferroptosis by examining morphological and biochemical characteristics. In contrast to other forms of cell death, lipid peroxidation is a crucial marker of ferroptosis. To investigate the level of ACE-mediated lipid peroxidation, two fluorescent probes, BODIPY-C11 and Liperfluo, were used in this assay. BODIPY-C11 staining revealed that ACE promoted lipid peroxidation more strongly than the ferroptosis inducers RSL3 and erastin did, and this effect was reversed by the iron chelator DFO and the lipid peroxidation scavenger Lip-1 (Fig. 3a, b, and Supplementary Fig. 3a–c). Moreover, ACE significantly increased the level of intracellular lipid peroxidation in a concentration-dependent manner (Fig. 3c and Supplementary Fig. 3d–i). In addition, ACE generally elevated lipid peroxidation levels in other colorectal cancer cells, including drug-resistant colorectal cancer cells (Supplementary Figs. 3j–l, and 4a–d). In addition, we detected a significant increase in the intracellular level of MDA, a cytotoxic product produced by lipid peroxidation metabolism (Fig. 3d and Supplementary Fig. 5a). Moreover, transmission electron microscopy revealed smaller mitochondria, increased membrane density, and fewer cristae, which indicate the morphological signature of ferroptotic cells (Fig. 3e). Additionally, ACE dose-dependently disrupted mitochondrial function, concomitant with significant inhibition of basal and maximal cellular respiration (Fig. 3f and Supplementary Fig. 5b, c). These results indicate that ACE treatment significantly increases the accumulation of lipid peroxides, subsequently inducing ferroptosis in colorectal cancer. Taken together, these data demonstrate that ACE induces canonical ferroptosis.

To detect free intracellular ferrous ions (Fe^2+^), the fluorescent probe (FerroOrange) and Ferrous Ion Content Assay Kit were used. As shown in Fig. 3g, h and Supplementary Fig. 5d–k, ACE treatment resulted in a significant dose-dependent increase in Fe^2+^ in RKO and HCT116 cells, and DFO, but not Lip-1, significantly reversed the effect of ACE on increasing Fe^2+^ levels. Surprisingly, compared with the ferroptosis inducers RSL3 and erastin, ACE significantly increased the intracellular Fe^2+^ levels (Fig. 3g and Supplementary Fig. 5e). Moreover, the multiomics results revealed that the most significantly upregulated gene was HO-1, which is associated with iron metabolism (Supplementary Fig. 2i). This finding was subsequently confirmed by Western blot experiments (Fig. 3i and Supplementary Fig. 6a). When free intracellular ferrous ions accumulate in the cytoplasm, ROS are generated via the Fenton reaction, subsequently leading to ferroptosis in tumor cells. As shown in Fig. 3j and Supplementary Fig. 6b–d, ROS levels increased significantly after ACE treatment and caused cell death, whereas the ROS inhibitors N-acetyl-L-cysteine (NAC) and GSH reversed ACE-induced cell death (Fig. 3k and Supplementary Fig. 6e). These results suggest the important role of Fe^2+^ in ferroptosis induced by ACE (noncanonical ferroptosis). Taken together, these findings indicate that ACE induces ferroptosis via both canonical and noncanonical pathways.

### ACE suppresses colorectal tumor growth in vivo

**Fig. 4:**
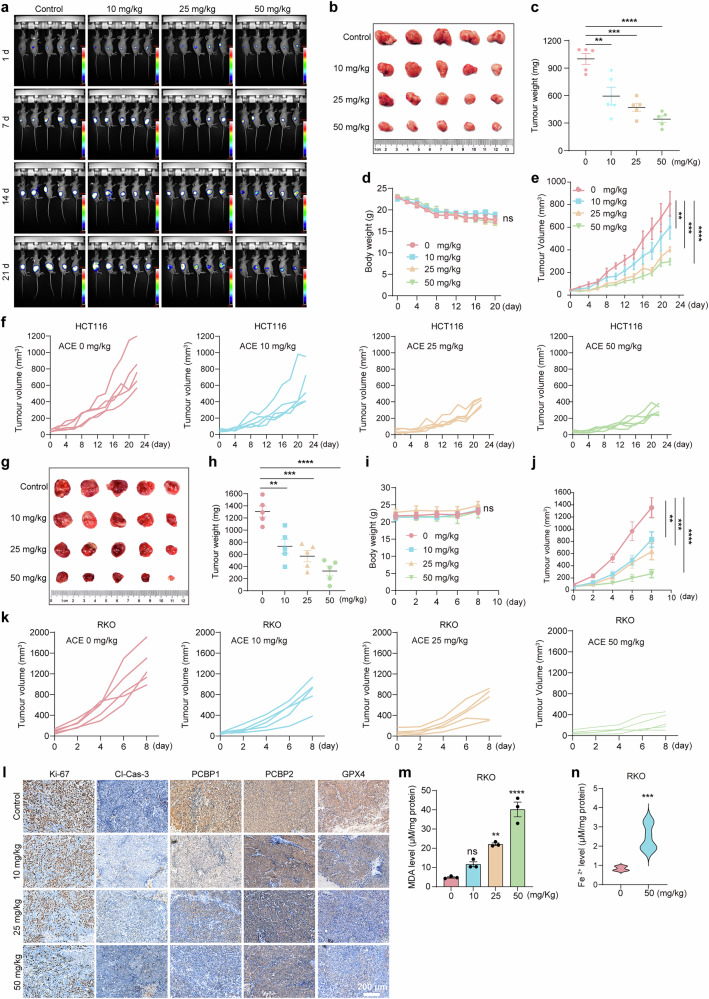
ACE induces ferroptosis in vivo. **a** Bioluminescence images of HCT116-luc tumors taken every week. **b, g** Xenograft tumor images of HCT116-luc (**b**) and RKO (**g**) fully grown tumors versus residual tumors treated with ACE (10, 25, and 50 mg/kg). **c**, **h** Tumor weights of (**b**) and (**g**). (n = 5, error bars represent SEM, one-way ANOVA). **d**, **i** Mouse body weight. (n = 5, error bars represent SEM, two-way ANOVA). **e**, **j** Tumor volume curves showing mice treated orally with ACE (10, 25, or 50 mg/kg) and measured every 2 days. (n = 5, error bars represent SEM, two-way ANOVA). **f**, **k** Tumor volume curves for each mouse in different treatment groups corresponding to (**e**) and (**j**), respectively. **l** Immunohistochemistry (IHC) images of Ki-67, cleaved caspase 3 (Cl-Cas-3), PCBP1, PCBP2, and GPX4 in corn oil- or ACE-treated (10, 25, or 50 mg/kg) HCT116-luc mice. Scale bar, 200 μm. **m** Quantification of tumor MDA levels in RKO mice after ACE (10, 25, or 50 mg/kg) treatment for 22 days. (n = 3, error bars represent SEM, one-way ANOVA). **n** Quantification of tumor Fe^2+^ levels in the corn oil or ACE (50 mg/kg) groups. (n = 3, error bars represent SEM, two-tailed unpaired Student’s t test). **P < 0.01, ***P < 0.001, ****P < 0.0001; ns not significant

Although ACE significantly inhibited GPX4 and elevated Fe^2+^ in colorectal cancer cells, thereby inducing ferroptosis, the tumor cytotoxicity of this compound in vivo is unknown. To verify the potential in vivo antitumor activity of ACE, mice subcutaneously inoculated with HCT116-luc tumors were treated with either corn oil or ACE orally once daily for 22 days (Supplementary Fig. 7a). The tumor fluorescence intensities in the 25 and 50 mg/kg groups were 57.5% and 36.95% of the control group, respectively (Fig. 4a and Supplementary Fig. 7b). In addition, treatment with ACE at 25 and 50 mg/kg significantly reduced tumor size, volume, and weight compared to the control group (Fig. 4b–f). There was no significant difference in weight among the different groups, and the immunohistochemical results also revealed no obvious toxicity to the heart, liver, spleen, lung, or kidney (Fig. 4d and Supplementary Fig. 7c). These results showed that ACE treatment significantly inhibited HCT116 tumor growth in mice. We subsequently seeded RKO cells into nude mice and administered ACE when the tumor volume approached 50 mm^3^. Similar to its efficacy at the cellular level, ACE was more effective in RKO tumor-bearing mice than in HCT116 mice (Fig. 4g–k).

Immunoblotting analysis of tumor tissues revealed that the number of Ki67-positive cells in ACE-treated tumors was significantly lower than that in the control group. Furthermore, ACE did not activate caspase-3, suggesting that ACE suppressed mouse tumor growth independent of apoptosis (Fig. 4l and Supplementary Fig. 7d). To determine whether ACE induced ferroptosis in tumor tissues, we measured the levels of the lipid peroxidation products MDA and Fe^2+^ and found that ACE elevated the levels of MDA and increased the accumulation of Fe^2+^ in mouse tumor tissues (Fig. 4m, n and Supplementary Fig. 7e, f). These results suggest that ACE induces ferroptosis in tumor cells and effectively inhibits colorectal cancer.

### ACE elevates Fe^2+^ levels independent of HO-1

Previous studies have suggested that elevated HO-1 may be a source of intracellular Fe^2+^.^32^ To elucidate the mechanism by which ACE elevates Fe^2+^, we further determined whether ACE increased intracellular Fe^2+^ by upregulating HO-1. Unexpectedly, ACE treatment significantly increased the intracellular Fe^2+^ levels within 12 min, but western blot analysis revealed that ACE did not upregulate HO-1 expression within 1 h (Supplementary Fig. 8a–d). Moreover, we cotreated cells with ACE and Znpp, an inhibitor of HO-1. However, no significant difference in cell viability was found, suggesting that ACE did not induce ferroptosis by activating the enzymatic activity of HO-1 (Supplementary Fig. 8e, f). Furthermore, ZnPP did not reverse the increase in Fe^2+^ levels induced by ACE (Supplementary Fig. 8g, h). This conclusion was further confirmed by using hemin, an agonist of HO-1 enzymatic activity (Supplementary Fig. 8g, i, j). Moreover, the changes in the ROS levels were not reversed by ZnPP or hemin (Supplementary Fig. 9a–d). These results indicate that ACE increases intracellular Fe^2+^ levels independently of HO-1. In addition, we found that cells appeared green after ACE treatment, suggesting that elevated intracellular Fe^2+^ plays an important role in ferroptosis induced by ACE (Supplementary Fig. 9e).

### Downregulation of PCBP1/2 mediates Fe^2+^ release and induces ferroptosis

**Fig. 5:**
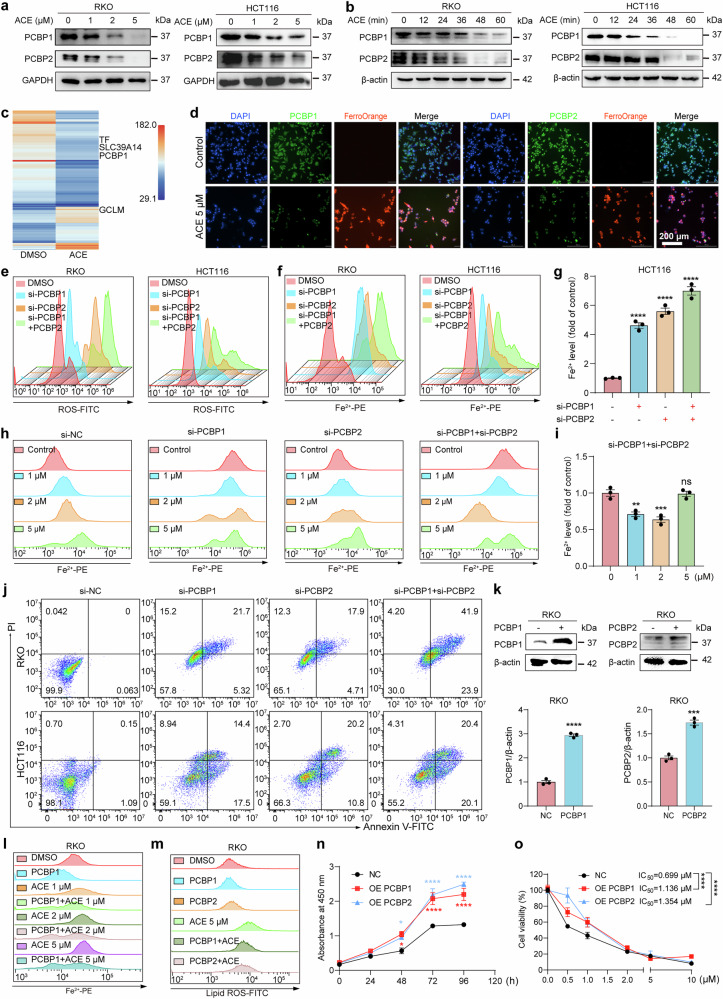
PCBP1/2 mediates ACE-induced Fe^2+^ accumulation and ferroptosis. **a, b** Western blot analysis of PCBP1 and PCBP2 protein levels in RKO and HCT116 cells after treatment with dose-dependent (**a**) or time-dependent (**b**) ACE. **c** Heatmap showing DARTS in the total protein of RKO cells treated with ACE for 1 h. Ferroptosis-related genes were labeled (fold change ≥1.2, unique peptides ≥2). **d** PCBP1/2 and Fe^2+^ levels in RKO cells were detected after ACE treatment for 24 h. The nuclei were stained with DAPI (blue), PCBP1/2 (green) was stained with a fluorescence-conjugated PCBP1/2 antibody, and Fe^2+^ was stained with FerroOrange (red) (n = 3 wells of a 12-well plate from one representative experiment; scale bars, 200 μm). **e, f** ROS levels (**e**) and Fe^2+^ levels (**f**) in PCBP1- or PCBP2-knockdown RKO and HCT116 cells were analyzed via flow cytometry. **g** Quantification of (**f)**. (n = 3, error bars represent SEM, one-way ANOVA). **h, i** Flow cytometry analysis of Fe^2+^ levels in ACE-treated (1, 2, 5 μM, 24 h) PCBP1- or PCBP2-knockdown RKO cells, and the Fe^2+^ levels were quantified in (**i**). (n = 3, error bars represent SEM, one-way ANOVA). **j** Cell death analysis of PCBP1- or PCBP2-knockdown RKO and HCT116 cells via flow cytometry. **k** Western blot showing the overexpression efficiency of PCBP1 and PCBP2 in RKO cells, and the quantitative results are shown. (n = 3, error bars represent SEM, unpaired two-tailed Student’s t test). **l, m** Flow cytometry analysis of Fe^2+^ levels (**l**) and lipid ROS levels (**m**) in ACE-treated PCBP1- or PCBP2-overexpressing RKO cells. **n** Cell proliferation analysis of OE NC, OE PCBP1, and OE PCBP2 RKO cells (n = 3, SEM, two-way ANOVA). **o** Cell viability analysis showing the dose-dependent toxicity of ACE (0.5, 1, 2, 5, and 10 μM) in OE NC, OE PCBP1, and OE PCBP2 RKO cells via a CCK-8 assay. (n = 3, SEM, two-way ANOVA). *P < 0.05, **P<0.01,  ***P<0.001,  ****P < 0.0001; ns not significant

To explore the potential mechanism by which ACE elevates Fe^2+^ levels, iron metabolism proteins associated with ACE were screened by Western blot based on proteomic results (Fig. 5a, b and Supplementary Fig. 10a–c). Furthermore, we used drug affinity responsive target stability (DARTS), a technique for detecting the binding of small-molecule drugs to their target proteins, to explore potential target proteins of ACE. DARTS predicted 240 potential target proteins (intensity ratio ≥ 1.2), and four genes, namely, TF, GCLM, PCBP1, and SLC39A14, were directly associated with ferroptosis (Fig. 5c). Notably, PCBP1 or PCBP2, which have similar functions in the regulation of iron metabolism, were enriched in two assays. Consistent with the multiomics results, PCBP1 and PCBP2 proteins were significantly downregulated in a dose-dependent and time-dependent (within 1 h) manner (Fig. 5a, b and Supplementary Fig. 10d–i). Therefore, we speculate that ACE may increase the release of Fe^2+^ through PCBP1 and PCBP2.

To verify the relationship between the elevated Fe^2+^ level of ACE and PCBP1/2, we performed immunofluorescence staining and found that PCBP1 and PCBP2 were significantly negatively correlated with intracellular Fe^2+^ (Fig. 5d and Supplementary Fig. 11a). We next designed a specific small interfering RNA (siRNA) to mimic the pharmacological inhibition of PCBP1/2 by ACE (Supplementary Fig. 11b, c). Interestingly, knockdown of PCBP1 and PCBP2 significantly elevated Fe^2+^ and ROS levels (Fig. 5e–g and Supplementary Fig. 11d, e). Furthermore, ACE did not significantly increase Fe^2+^ levels in cells lacking PCBP1/2 (Fig. 5h, i and Supplementary Fig. 11f–h). Moreover, we observed extremely poor cell status after knockdown, and the flow cytometry results indicated increased cell death in colorectal cancer cells (Fig. 5j and Supplementary Fig. 12a). These results suggest that ACE may induce ferroptosis by increasing Fe^2+^ and ROS via PCBP1 and PCBP2. As expected, the overexpression of PCBP1 and PCBP2 significantly inhibited the ability of ACE to increase lipid peroxidation and Fe^2+^, which further confirmed our speculation (Fig. 5k–m and Supplementary Fig. 12b–e). Moreover, cell proliferation was significantly promoted, and the cytotoxicity of ACE was significantly inhibited when PCBP1 and PCBP2 were overexpressed in RKO cells (Fig. 5n, o). These results suggest that ACE induces ferroptosis in colorectal cancer through PCBP1 and PCBP2.

### Binding of ACE to PCBP1/2 induces ferroptosis

**Fig. 6:**
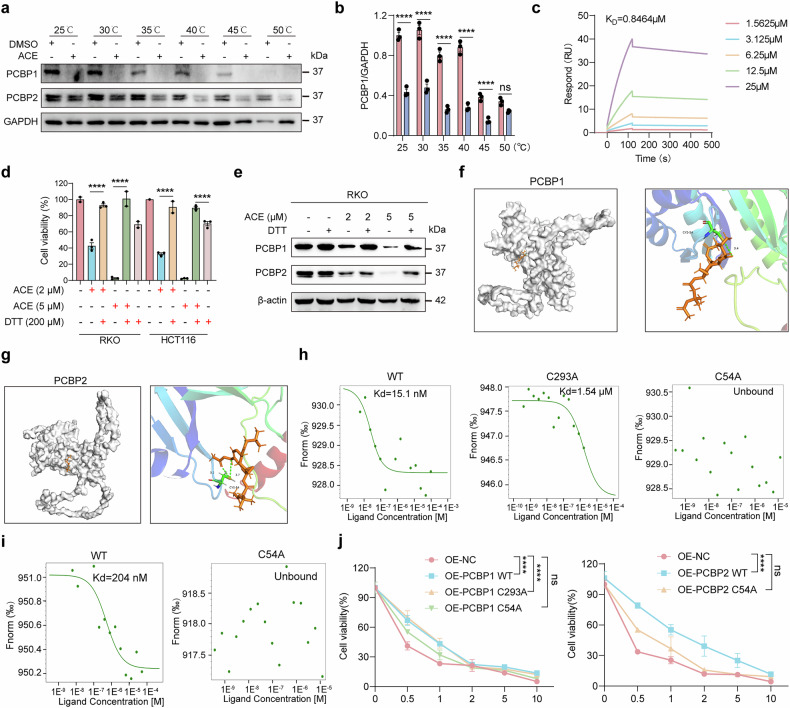
ACE directly binds to PCBP1/2. **a**, **b** CETSA showing the effect of ACE on the thermal stability of the PCBP1/2 protein in RKO cells. (n = 3, error bars represent SEM, two-way ANOVA). **c** Surface plasmon resonance (SPR) assay to determine the affinity between PCBP1 and ACE. **d, e** RKO cells were treated with ACE (2 or 5 μM) with or without the cysteine-rich thiol donor DTT for 24 h, after which cell viability (**d**) and protein levels (**e**) were detected. (n = 3, error bars represent SEM, one-way ANOVA). **f, g** Molecular modeling simulation of ACE docked on AlphaFold-predicted PCBP1 (**f**) (AF-Q15365-F1) and PCBP2 (**g**) (AF-Q15366-F1). **h, i** MST assay showing the binding of predicted binding site mutants, wild-type PCBP1 (**h**) or PCBP2 (**i**) to ACE. **j** Cell viability analysis showing the dose-dependent toxicity of ACE (0.5, 1, 2, 5, and 10 μM) in OE NC, OE PCBP1-C54A, and OE PCBP2-C54A RKO cells via a CCK-8 assay. (n = 3, error bars represent SEM, two-way ANOVA). ****P < 0.0001; ns not significant

To investigate whether ACE directly binds to PCBP1 and PCBP2, we used a cellular thermal shift assay (CETSA) to detect whether the thermal stability of the proteins changed after ACE treatment. As shown in Fig. 6a, b, the PCBP1 and PCBP2 proteins were less thermally stable after ACE treatment, which was consistent with the results of DARTS. Furthermore, we determined the affinity between PCBP1 and ACE via surface plasmon resonance (SPR), and the results revealed that ACE bound directly to PCBP1 with a KD value of 0.8464 μM (Fig. 6c). Additionally, ACE dissociates slowly from the PCBP1 protein, suggesting that ACE binds to the PCBP protein in a more stable manner (Fig. 6c). In addition, DTT, a cysteine-rich thiol donor, could compete for cysteine-dependent ACE binding to PCBP1/2. As shown in Fig. 6d, e, the downregulation of PCBP1/2 and the increase in cytotoxicity caused by ACE were partially reversed by the addition of excess DTT, indicating that ACE binds directly to cysteine residues of PCBP. We then used Molecular Operating Environment (MOE) software to model the covalent binding of ACE with PCBP1/2 via its cysteine residues. Docking simulations were performed to explore the binding mode of ACE to each cysteine residue site of PCBP1 (AlphaFold ID AF-Q15365-F1) and PCBP2 (AlphaFold ID AF-Q15366-F1). As shown in Fig. 6f, g stable hydrogen bonds were formed between Cys54 and the backbone of ACE, suggesting that Cys54 is a possible binding site for both PCBP1 and PCBP2. In addition, Cys293 is another possible binding site for PCBP1. To further confirm the direct binding of ACE to PCBP1/2, we overexpressed wild-type PCBP1/2, the Cys54 mutant PCBP1, the Cys293 mutant PCBP1, and the Cys54 mutant PCBP2 with a GFP tag and subsequently analyzed direct binding via microtiter thermophoresis (MST). As expected, ACE bound strongly to PCBP1/2, with estimated Kd values of 4.64 nM and 204 nM. When Cys293 was mutated to alanine, its Kd became 325 times than that of wild-type PCBP1, whereas when Cys54 was mutated to alanine, ACE binding could not be detected in either PCBP1 or PCBP2 (Fig. 6h, i). Consistently, we found that the cytotoxicity of ACE was reduced in RKO cells overexpressing PCBP1/2 C54A (Fig. 6j). These results suggest that ACE elevates Fe^2+^ levels to induce ferroptosis in colorectal cancer through direct binding to PCBP1 and PCBP2.

### ACE acts as a natural class II ferroptosis-inducing agent

**Fig. 7:**
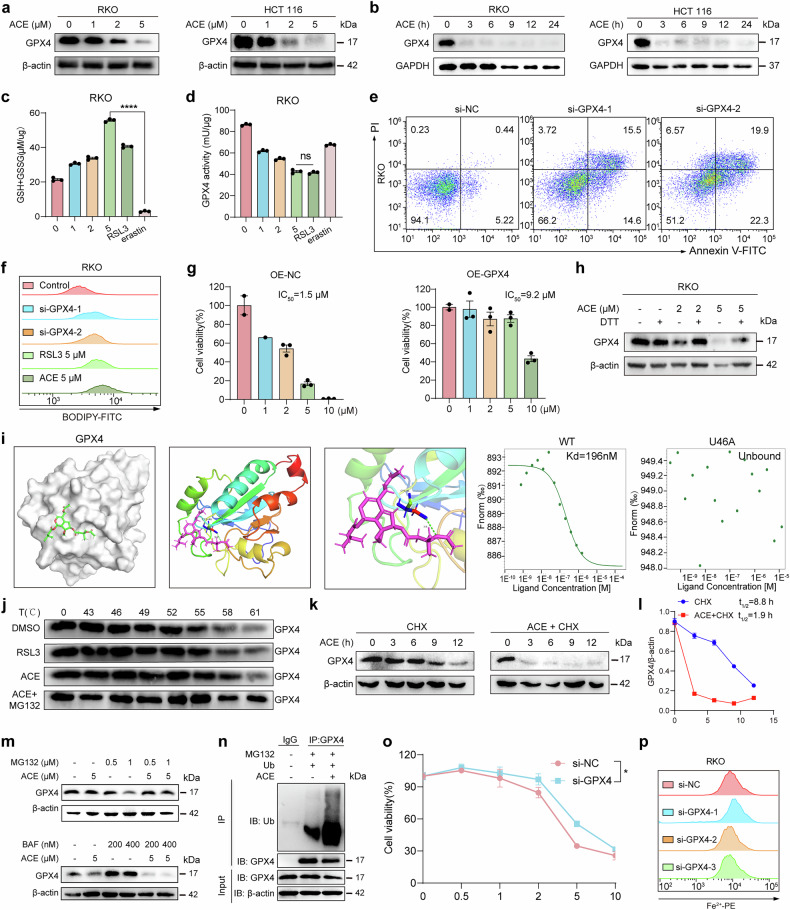
ACE induces GPX4 depletion for ferroptosis. **a, b** Western blot analysis of GPX4 expression in RKO and HCT116 cells after treatment with dose-dependent (**a**) or time-dependent (**b**) ACE. **c, d** Measurement of GSH levels (**c**) and GPX enzyme activity (**d**) in RKO cells after treatment with ACE (1, 2, or 5 μM) for 24 h. (n = 3, error bars represent SEM, one-way ANOVA). **e, f** Flow cytometry analysis of cell death (**e**) and lipid peroxidation (**f**) in GPX4-knockdown or WT RKO cells. **g** Dose-dependent toxicity of ACE in RKO cells overexpressing GPX4, and CCK-8 assays were used to measure cell viability. (n = 3, error bars represent SEM). **h** Western blot analysis of RKO cells treated with ACE (2 or 5 μM) with or without DTT for 24 h. **i** Molecular docking of ACE to GPX4 (6nh3). GFP-tagged wild-type or U46 disruptive mutant GPX4 proteins were maintained at constant concentrations and fluorescence intensities, and ACE was diluted in a 1/2-fold gradient. The MST-on time of 1.5 s and dissociation constants K_d_ were determined. **j** Thermal stability analysis of GPX4 protein interactions with the indicated compounds from 43 °C to 61 °C. **k**, **l** CHX analysis of GPX4 abundance in RKO cells treated with or without ACE (5 μM) for different durations was performed by immunoblotting, and the GPX4 intensity was quantified in (**l**). (n = 3, error bars represent SEM, two-way ANOVA). **m** Immunoblotting was used to detect the degradation of GPX4 in RKO cells induced with lysosome (Baf) and proteasome (MG132) inhibitors. **n** IP assay showing the ubiquitin (Ub) modification of GPX4 in RKO cells treated with ACE (5 μM) for 1 h, after which MG132 was added and incubated for 3 h. **o** CCK-8 assay showing the dose-dependent toxicity of ACE in RKO cells transfected with si-NC or si-GPX4. **p** Flow cytometry analysis of Fe^2+^ levels in GPX4-knockdown RKO cells. *P < 0.05, ****P < 0.0001; ns not significant

As the upregulation of a series of antioxidant genes and factors was observed in the multiomics data, we examined the changes in the expression of several antioxidant genes. Consistent with the multiomics results, ACE significantly upregulated the expression of nuclear factor erythroid derived 2-like 2 (Nrf2) and its downstream target genes (e.g., SLC3A2, SLC7A11, and GCLM), ferroptosis suppressor protein 1 (FSP1), and dihydroorotate dehydrogenase (DHODH) (Supplementary Fig. 13a, b). Moreover, we found that the expression of GPX4, a core protein of the ferroptosis defense mechanism, was significantly downregulated (Fig. 7a, b and Supplementary Fig. 13c, d). Inactivation of GPX4 can occur through two processes: depletion of intracellular glutathione (GSH) or direct targeting of GPX4. As shown in Fig. 7c and Supplementary Fig. 2h and13e, ACE treatment increased the levels of GSH and cystine in RKO cells. Furthermore, ACE rapidly increased ROS levels and inhibited GPX enzyme activity, indicating that ACE, like RSL3, may bind directly to GPX4 (Fig. 7d and Supplementary Fig. 13f–h). As anticipated, the lack of GPX4 significantly promoted lipid peroxidation accumulation and cell death (Fig. 7e, f and Supplementary Fig. 13i–m). However, GPX4 overexpression increased the IC_50_ value of ACE in RKO cells (Fig. 7g and Supplementary Fig. 13n, o). Excess DTT reversed the downregulation of GPX4, confirming the binding between ACE and the cysteine residues of GPX4 (Fig. 7h and Supplementary Fig. 14a, b). As shown in Fig. 7i, we similarly performed molecular docking simulations and found that the active site, selenocysteine (U46), was the most favorable site for the covalent binding of GPX4 by ACE (Protein Data Bank ID 6NH3). Additionally, the thermal stability of the GPX4 protein increased upon treatment with ACE and RSL3 (positive control), indicating that ACE can bind to and stabilize GPX4 (Fig. 7j and Supplementary Fig. 14c). Finally, we performed MST assays of GFP-tagged GPX4 in the wild-type and disruptive mutant U46. The Kd value for ACE binding to the GPX4 protein was estimated to be 196 nM (Fig. 7i). However, once U46 was mutated to alanine, the binding between GPX4 and ACE was lost, which further diminished the cytotoxicity of ACE (Fig. 7i and Supplementary Fig. 14d, e). These results suggest that ACE inactivates GPX4 through direct binding to U46 in GPX4, triggering lipid peroxidation and subsequent ferroptosis.

Next, we investigated the mechanisms by which ACE triggered GPX4 protein degradation. Western blot and real-time PCR results revealed that ACE downregulated not only GPX4 expression but also its mRNA levels (Fig. 7a, b and Supplementary Figs. 13c, S14f). Here, we explored mainly the posttranslational regulation of GPX4 since its protein levels decrease more rapidly than its RNA levels (Fig. 7b and Supplementary Figs. 13d, 14g). As shown in Fig. 7k, l, in combination with the protein translation inhibitor cycloheximide (CHX), the half-life of GPX4 in ACE-treated cells was faster than that in untreated cells, suggesting that ACE degraded GPX4 mainly at the protein level. In addition, Western blot experiments demonstrated that the proteasome inhibitor MG132 reversed the degradation of GPX4 by ACE but not by CQ (Fig. 7m and Supplementary Fig. 14h). The immunofluorescence results also revealed that the number of lysosomes did not increase (Supplementary Fig. 14i). Moreover, IP assays revealed that ACE increases the ubiquitination of GPX4 (Fig. 7n). These results suggest that ACE mediates the degradation of GPX4 through the ubiquitin-proteasome pathway and induces ferroptosis in colorectal cancer cells.

Finally, the cytotoxicity of RSL3 disappeared, whereas the cytotoxicity of ACE was partially reduced when GPX4 was knocked down since RSL3 did not increase Fe^2+^ (Fig. 7o, p and Supplementary Fig. 14j, k). These findings explain why ACE was more cytotoxic than RSL3 in oxaliplatin-resistant HCT116 cells (Supplementary Fig. 1e). Furthermore, PCBP1, PCBP2, and GPX4 synergistically promoted ferroptosis in colorectal cancer cells (Supplementary Fig. 14l, m). As shown in Fig. 4l, GPX4, PCBP1, and PCBP2 protein levels were reduced in tumor tissues in a dose-dependent manner, which was consistent with the results of the in vitro experiments. These results emphasize the effectiveness of the dual mechanism of ACE in inducing ferroptosis in colorectal cancer cells, which inactivates GPX4 and downregulates PCBP1/2 to release Fe^2+^.

### Protein expression levels of PCBP1/2 and GPX4 in human tumor tissue

**Fig. 8:**
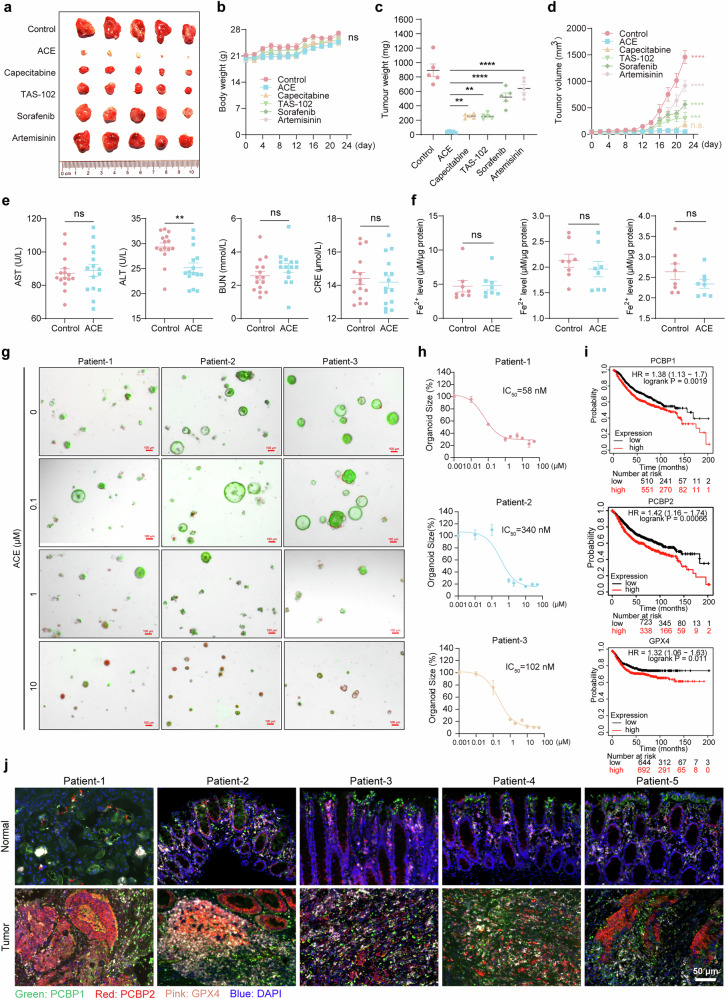
Targeting PCBP1/2 as a potential strategy for tumor suppression. **a-d** Xenograft tumor images (**a**), tumor weights (**b**), body weights (**c**) and tumor volume curves (**d**) of RKO fully grown tumors versus residual tumors treated with ACE (50 mg/kg), capecitabine (500 mg/kg), TAS-102 (150 mg/kg), sorafenib (30 mg/kg), and artemisinin (50 mg/kg). (n = 5, error bars represent SEM, two-way ANOVA). **e, f** Quantitative analysis of ALT, AST, BUN, and CRE levels (**e**) and Fe^2+^ levels (**f**) in mice treated orally with ACE (50 mg/kg). **g, h** Calcein/PI staining (**g**) and organoid size (**h**) showing colorectal cancer organoids treated with ACE (0.01, 0.1, 1, 2, 5, 10, 25, or 50 μM) for 6 d. Scale bars, 100 μm. **i** Survival curves of CRC patients based on the expression of PCBP1, PCBP2, and GPX4. Scale bars, 50 μm. **j** The expression levels of PCBP1, PCBP2, and GPX4 in normal and tumor tissues of colorectal cancer patients were detected via triple-color fluorescence staining IHC. **P < 0.01, ****P < 0.0001; ns not significant

To validate the potential of the above models for clinical tumor treatment, we compared the tumor inhibitory effects of ACE with those of clinical first-line drugs and ferroptosis-positive drugs in xenograft models. We found that the tumor inhibitory effect of ACE was significantly greater than that of the ferroptosis-positive drugs sorafenib and artemisinin and even better than that of the clinical first-line drugs capecitabine and TAS-102, since ACE elevated Fe^2+^ and reduced PCBP2 and GPX4 significantly better than the other treatments did (Fig. 8a–d, Supplementary Fig. 15a–d). Although ACE induces ferroptosis in tumor cells by targeting PCBP1/2 and GPX4, previous studies have shown that ferroptosis may cause acute liver injury and acute kidney injury. To evaluate the safety of ACE for tumor therapy, we further conducted acute toxicology experiments in which mice were orally administered ACE (50 mg/kg) daily for 10 days. As shown in Supplementary Table 1, there were no significant changes in the blood parameters of mice, such as red and white blood cell counts or platelet counts; indicators of liver and kidney injury, such as ALT, AST, BUN, Cr, etc., were within the normal range (Fig. 8e). In addition, we examined the Fe^2+^ levels in the heart, liver, and kidney tissues of the mice and found that there was no significant difference between the heart, liver, and kidney tissues of the ACE-treated mice and those of the control group (Fig. 8f). These results indicate that ACE at therapeutic doses has no significant risk of ferroptosis induction in normal tissues and that the clinical application of ACE provides a solid basis for translational medicine.

We constructed a colorectal cancer organoid model to evaluate the sensitivity of ACE. After being cultured with various concentrations of ACE (0.01, 0.1, 1, 5, 10, 25, or 50 μM) for six days, the size of the live cells, represented by green fluorescence, significantly decreased, and the number of dead cells, represented by red fluorescence, significantly increased, indicating that the growth of the organoids was significantly inhibited, as shown in Fig. 8g. The sensitivity of the organoids was measured via the area method, and the IC_50_ values of ACE were found to be 58, 148, and 340 nM for the three organoid models (Fig. 8h). The analysis of overall survival in colorectal cancer patient samples revealed that patients with high levels of GPX4, PCBP1, and PCBP2 had significantly worse overall survival (Fig. 8i). Additionally, we investigated the protein expression of PCBP1, PCBP2 and GPX4 in colon cancer patients via immunofluorescence staining (Fig. 8j and Supplementary Fig. 15e, f). Consistent with our results, the expression levels of these proteins were significantly higher in tumor tissues than in paracancerous tissues from patients with colon adenocarcinoma (COAD) or rectal adenocarcinoma (READ) (Human Protein Atlas) (Supplementary Fig. 15g–i). Most importantly, PCBP 1/2 and GPX4 are highly expressed in most human cancers (Human Protein Atlas) (Supplementary Fig. 15j–k). These findings suggest that PCBP1 and PCBP2 have potential clinical significance as biomarkers for cancer diagnosis.

## Discussion

Although some progress has been made in the use of traditional chemotherapeutic drugs for anticancer therapy, a series of problems, such as drug resistance, recurrence and metastasis resulting from the induction of tumor cell apoptosis, have prompted us to develop drugs that induce other modes of tumor cell death.^33–36^ Ferroptosis is an iron-dependent mode of cell death that, unlike apoptosis and autophagy, induces cell death mainly by catalyzing lipid peroxidation of unsaturated fatty acids highly expressed on the cell membrane, which also makes ferroptosis promising for overcoming the disadvantage of apoptosis in tumor therapy.^37^ Moreover, the survival of drug-resistant cancer cells is dependent on GPX4, which plays a key role in cell death (ferroptosis) and is considered a novel target of anticancer drugs.^38,39^ Currently, many small-molecule drugs have shown encouraging effects in tumor therapy by inducing ferroptosis.^11^ Here, we screened ACE, a natural phytochemical isolated from vetiver roots, and demonstrated for the first time that ACE significantly inhibits colorectal cancer growth, metastasis, and drug resistance through dual ferroptosis mechanisms. Previous studies on ACE have focused mainly on isolation but less on specific antitumor mechanisms. Our key findings demonstrate that ACE exerts potent antitumor effects by simultaneously targeting PCBP1/2 (iron chaperones) and GPX4 (a key ferroptosis defense enzyme). On the one hand, ACE can rapidly increase Fe^2+^ by directly binding to PCBP1/2 and then increasing nonenzymatically catalyzed lipid peroxidation. On the other hand, ACE directly binds GPX4 to inactivate its enzymatic activity and degrades GPX4 through the ubiquitin pathway.

Although GPX4 inhibitors have made significant progress in inducing ferroptosis, as an increasing number of antioxidant systems are being discovered, it is still necessary to explore other mechanisms that induce ferroptosis. Similar to recent studies on dual ferroptosis inducers, the dual-targeting strategy is consistent with the emerging paradigm of combinatorial targeting to overcome compensatory mechanisms.^32^ Furthermore, artemisinin derivatives have demonstrated efficacy in eradicating oxaliplatin-resistant CRC cells through ferroptosis, thereby underscoring the potential of ferroptosis in combating drug resistance.^11,40^ Currently, the upregulation of HO-1 is one of the strategies used to increase Fe^2+^ levels, but the role of HO-1 in ferroptosis has been controversial. Excessive HO-1 activation has been proposed to be cytotoxic, but this is based on insufficient ferritin buffering capacity.^32^ Moreover, the Nrf2/HO-1 axis inhibits ferroptosis.^41^ In our study, ACE effectively induced ferroptosis in tumor cells by increasing the intracellular Fe^2+^ concentration. However, we found that ACE-induced HO-1 is largely a phenomenon but not a determinant for increasing Fe^2+^ to induce ferroptosis, since it was not rapidly increased in our study and could not be modulated by hemin or Znpp.

The poly(C)-binding protein (PCBP) family consists of four members that act as RNA-binding proteins and were originally found to be responsible for target gene transcription, mRNA stability, and translation.^42^ PCBP has been reported to bind to Fe^2+^ and is also known as an iron chaperone. Fe^2+^ was initially transferred from DMT1 to PCBP2, and PCBP2 is responsible for transferring Fe^2+^ to ferritin for storage. In addition to ferritin, PCBP1 and PCBP2 can also deliver iron to proteins that require nonheme iron as a cofactor or bind to ferroportin (Fpn) to remove excess Fe^2+^ from cells.^43–45^ PCBP1 and PCBP2 are involved in all aspects of import, export, storage, and utilization and are essential for maintaining cellular Fe^2+^ levels. Therefore, targeting PCBP has become a rapid and efficient pathway for increasing Fe^2+^ levels. Our proteomic analysis revealed that the PCBP protein content was significantly reduced in the ACE-treated group, and the DARTS results also indicated that PCBP may be a target protein of ACE. Therefore, we identified a PCBP1/2-targeting inhibitor, ACE. The results of the present study suggest that targeting PCBP1/2 is critical for increasing the level of intracellular free Fe^2+^, thereby contributing to the induction of ferroptosis. Furthermore, this pathway may be beneficial for the rapid regulation of cellular iron homeostasis since the activation of Nrf2 and subsequently HO-1 increases Fe^2+^ or because the activation of NCOA4, which induces ferritinophagy to further release Fe^2+^ from FTH, seems to be a relatively slow process. BAY 11–7085 increases Fe^2+^ through the upregulation of HO-1 but is limited by Nrf2 feedback inhibition.^12^ In addition, NCOA4-mediated ferroptosis promotes ferritin deposition induced by melanin but not RSL3 in HeLa cells,^46^ suggesting a limitation of the strategy to induce ferroptosis by releasing ferrous ions via NCOA4 or HO-1. In contrast, targeting PCBP1/2, an Fe^2+^ delivery vehicle, is more direct and effective.

The lethal accumulation of lipid peroxides is a major feature of ferroptosis and involves the confrontation between intracellular ferroptosis execution and the ferroptosis defense system. Ferroptosis occurs when the cellular activity promoting ferroptosis significantly exceeds the antioxidant buffering capacity provided by the ferroptosis defense system. Thus, most cells resist ferroptosis through complex antioxidant systems. For example, FSP1 and DHODH neutralize lipid peroxy radicals through CoQ10, Nrf2 suppresses ferroptosis through downstream target genes, and DHCR7 regulates ferroptosis by generating 7-DHC to clear ROS.^47,48^ However, GPX4, which converts lipid peroxides to lipid alcohols, has been the focus of recent developments in ferroptosis-inducing molecules.^2,9,49^ GPX4 is also strongly associated with acquired drug resistance and tumor metastasis. Currently, small-molecule inhibitors of GPX4 can be divided into two categories: direct targeted inactivation or indirect inhibition of cysteine uptake and GSH synthesis. For the latter, although FDA-approved sorafenib and salazosulfapyridine have been shown to induce ferroptosis through this pathway, tumor cells can compensate for cysteine and GSH through the SLC7A11 protein, ASC transporter system, and thiol antioxidants, resulting in limited therapeutic effects.^50^ Conventional GPX4 inhibitors are prone to resistance because of the upregulation of SLC7A11 or FSP1. Unlike traditional ferroptosis inducers (such as GPX4 inhibitors, such as RSL3), ACE overcomes resistance through a unique mechanism. ACE creates a “double whammy” by simultaneously increasing the Fe^2+^ levels (through inhibiting PCBP1/2) and blocking GPX4 function, significantly impairing the compensatory capacity of the cell, such as FSP1/CoQ10 and SLC7A11. Therefore, direct targeting and inactivation of GPX4 may be an effective strategy for bypassing adaptive mechanisms and drug resistance in cancer cells. Despite the potential side effects of GPX4 inhibitors, for example, GPX4 inactivation triggers acute renal failure and hepatic ischemia/reperfusion injury. However, we performed a drug safety evaluation, and ACE did not induce acute hepatic or renal injury or elevated ferrous ion levels in normal tissues.

The present study revealed the dual mechanism by which ACE induces ferroptosis by targeting PCBP1/2 and GPX4, but the following limitations and future research directions remain. The synergistic effects of lipid metabolism remain to be resolved: our study focused on the regulatory roles of PCBP1/2 and GPX4, but ACSL4 (acyl coenzyme A synthetase long-chain family member 4) and LOX (lipoxygenase) are highly expressed in colorectal cancer cells, which may synergistically amplify ACE-induced lipid peroxidation by increasing membrane incorporation and oxidation of polyunsaturated fatty acids (PUFAs). Future validation of the contribution of ACSL4/LOX to the action of ACE is needed. The mechanism of GPX4 regulation has not been fully elucidated: although it has been confirmed that ACE promotes ubiquitinated degradation of GPX4, the specific E3 ubiquitin ligases responsible for this process have not yet been identified. Although GPX4 protein degradation precedes its mRNA reduction, it remains unclear whether ACE additionally suppresses GPX4 transcription through epigenetic modifications (e.g., DNA methylation or histone acetylation).". Structural interaction studies need to be performed: although CETSA, MST, and SPR experiments confirmed the physical binding of ACE to PCBP1/2, the precise conformation of the binding site still needs to be resolved by cryo-EM (cryo-electron microscopy) or X-ray crystallography to guide structure-based drug optimization. The synergistic potential of immunotherapy can be explored: PCBP1 has been reported as a potential target for immunotherapy, but whether ACE-induced ferroptosis enhances the antitumor immune response through the release of damage-associated molecular patterns (DAMPs) remains to be verified. Immunologically sound mouse models can be constructed to assess the synergistic effect of ACE in combination with PD-1 inhibitors. Microenvironmental effects need to be systematically evaluated: previous studies have shown that ACE induces apoptosis by inhibiting HIF-1α,^51^, so whether hypoxia regulates ACE sensitivity through HIF-1α signaling deserves further investigation.

Taken together, our work demonstrated that ACE induces ferroptosis in tumor cells through a dual mechanism: on the one hand, ACE enhances intracellular Fe^2+^-induced ferroptosis by directly binding and degrading PCBP 1/2, and on the other hand, ACE induces GPX4 ubiquitination and degradation by directly binding to GPX4. Moreover, ACE showed strong antitumor effects in mouse colon cancer models and even in human colon cancer organoid models. The dual-target mechanism of ACE not only circumvents the problem of compensatory drug resistance of single-target inducers, but also achieves highly efficient and low-toxicity tumor-selective killing through the multi-targeting properties of natural products. We expect this dual mechanism to provide new strategies for the clinical management of colorectal cancer.

## Materials and methods

### Cell culture

Colorectal cancer cell lines (RKO, HCT116, DLD1, LOVO, SW620 and CT26), hepatocellular carcinoma cell lines (Hepa 1-6 and HCCLM3), gastric cancer cell line HGC-27, gallbladder cancer cell line GBC-SD and normal colonic epithelial cell line NCM460 were purchased from the Shanghai Institute of Cell Biology, Chinese Academy of Sciences (Shanghai, China). The pancreatic carcinoma cell line PANC-1, prostate adenocarcinoma cell line PC-3, breast cancer cell line (MCF7 and 4T1), and lung adenocarcinoma cell line NCI-H1975 were purchased from American Type Culture Collection (USA). The cells were cultured in RPMI-1640, DMEM, MEM F-12K, or McCoy’s 5 A medium (Meilunbio, China) supplemented with 10% FBS (Gibco, USA) and 1% antibiotics (Gibco, USA). All of the cells were incubated at 37 °C in an incubator with 5% CO^2^ and a humidified atmosphere.

### Animal experiments

Male nude mice, ~20 g and 6 weeks old, were purchased from Shanghai Southern Model Biotechnology (Shanghai, China). The animals were maintained in a specific pathogen-free environment at the Animal Laboratory Center of Shanghai University of Chinese Medicine. All animals were subjected to the same housing conditions, including the same temperature, humidity, and light cycle, to ensure the consistency of the experimental conditions. A standardized dietary regimen was used for all experimental animals to ensure consistent dietary composition and to avoid the potential influence of dietary factors on the experimental results. The study protocols and procedures were approved by the Animal Experimental Ethics Committee of Shanghai University of Chinese Medicine. Xenograft models were established to evaluate the effects of the drugs in vivo. Mice were subcutaneously inoculated with either RKO cells (5 × 10^6^ cells) or HCT116-luc cells (2 × 10^6^ cells). After subcutaneous inoculation of tumor cells, mice were randomly grouped according to tumor volume and body weight when the tumor volume reached 50 mm^3^. ACE was orally administered at doses of 0, 10, 25, and 50 mg/kg. Tumor size and fluorescence intensity were measured every 2–3 days.

### Cell transfection for gene overexpression and silencing

To achieve overexpression, full-length PCBP1, PCBP2, and GPX4 were inserted into the pcDNA3.1 vector. siRNAs for PCBP1, PCBP2, and GPX4 were purchased from GenePharma (Shanghai, China), and their sequences are shown in Supplementary Table 2. Cells were plated at a density of 3 × 10^5^ cells per well and cultured in six-well plates for 24 h before transfection. Transfection with siRNAs or expression plasmids was conducted using Lipofectamine 3000 Reagent (Invitrogen, Carlsbad, CA) following the manufacturer’s protocols. The cells were validated using Western blot and quantitative real-time PCR after 2–3 days.

#### Cellular thermal shift assay (CETSA)

For CETSA experiments, cells were collected, washed twice with PBS and centrifuged for 3 min at 400 × *g*. The cells were subsequently lysed with liquid nitrogen and centrifuged, after which the supernatant was collected. Aliquots were treated with the indicated compounds (100 μM) or DMSO for 30 min at room temperature. The sample was divided into 50 μL/tube, heated at a designated temperature range for 3 min, and cooled to room temperature. All samples were centrifuged, and the supernatants were diluted with 5× loading buffer and analyzed via Western blot.

#### Microscale thermophoresis (MST)

To verify whether ACE binds to wild-type or mutant PCBP1, PCBP2, and GPX4. A plasmid carrying the GFP target protein was constructed and subsequently expressed in 293T cells. Cell lysates were obtained after 48 h and mixed with different concentrations of ACE. The samples were subsequently analyzed via a MonolithTM NT.115 MST device (NanoTemper, Germany).

#### Molecular docking of ACE to PCBP1, PCBP2, and GPX4

The amino acid sequences of GPX4 (code: P36969), PCBP1 (code: Q15365), and PCBP2 (code: Q15366) were obtained from UniProt. The crystal structure of GPX4 (PDB id: 6NH3) was obtained from the RCSB Protein Data Bank (PDB, https://www.rcsb.org/). The 3D structures of PCBP1 and PCBP2 were predicted by Alph-Fold. The chemical structure of ACE (compound CID: 65717) was extracted from the PubChem database (https://pubchem.ncbi.nlm.nih.gov/) and minimized via Molecular Operating Environment (MOE) software. The protein structure was structurally corrected, protonated, unbound water molecules were removed, and the energy was optimized via the QuickPrep tool in MOE. The Site Finder tool in MOE was used to identify possible binding sites, and the cysteine residue was selected as the covalent docking site. Finally, the binding mode with the best docking score was selected as the final covalent binding structure.

### Cellular glutathione peroxidase activity assay

Glutathione peroxidase activity was measured according to the instructions provided with the Cellular Glutathione Peroxidase Assay Kit (Beyotime, China). Cells were treated with varying concentrations of ACE, and cell lysates were harvested after 24 h. Subsequently, 10 μL of cell lysate was placed in a 96-well plate and incubated with assay buffer for 15 min. Then, 2 μL of 62.5 mM NADPH, 2 μL of 75 mM GSH, and 1 μL of glutathione peroxidase were added to the assay buffer to deplete the GSSG in the samples. The activity of glutathione peroxidase was determined by measuring the change in absorbance at 340 nm over a period of 5–10 min after the addition of 10 μl of 30 mM peroxidizing agent (t-Bu-OOH).

#### Drug screening, cell viability, and cell death assays

For drug screening, compounds of unknown biological activity were purchased from Selleck Chemicals, and RKO cells were treated with 10 μM compound for 24 h. A Cell Counting Kit-8 (APExBIO, USA) was then used to detect cell viability.

Cells were plated at 5 × 10^3^ cells per well in 96-well plates (Corning, USA) and incubated overnight to allow them to adhere. Cells were pretreated with the indicated inhibitors prior to further treatment or treated directly with the specified compounds at the designated concentrations for the indicated times. A Cytation 5 Cell Imaging Reader (BioTek, USA) was used to quantify the absorbance at 450 nm (cellular formazan). Relative cell viability (%) was calculated as follows: (absorbance of sampl − background)/(absorbance of control sample − background) × 100. A fitted curve of cell viability was generated via GraphPad Prism 9.0.

In addition, cell death was detected via a Calcein/PI Live/Dead Viability/Cytotoxicity Assay Kit (Beyotime, China). The assays were performed with steps similar to those described in the product manual, and the number of propidium iodide-positive cells was calculated via the high-concentration imaging system Operetta CLS (PerkinElmer, USA).

#### Cell growth assay

An EdU Cell Proliferation Kit (Beyotime, China) was used to assess cell proliferation by detecting DNA synthesis in cells. Cells were seeded in 12-well plates at 2 × 10^6^ cells per well and incubated with ACE in triplicate wells. Prewarmed EdU (20 μM, 37 °C) was added to the medium and incubated for 2 h to complete the EdU labeling. The cells were then fixed with 4% paraformaldehyde (PFA) and permeabilized with 0.3% Triton X-100. Finally, click reaction solution was added, and the mixture was incubated for 30 min (25 °C) to complete fluorescent labeling. EdU-positive cells were quantified via Operetta CLS (PerkinElmer, USA).

Colony formation assays were designed to evaluate the effects of ACE on the long-term proliferative ability of cells. Briefly, a total of 1000 cells were plated in 6-well plates. The indicated concentrations of ACE were added to the media, which were subsequently allowed to form colonies for 10–14 days. Colonies were fixed with 4% paraformaldehyde (PFA) and stained with 0.5% crystal violet (Beyotime, China). A Cytation 5 (BioTek, USA) was used for imaging and counting.

#### Apoptosis, cell cycle progression, and reactive oxygen species (ROS) analysis

After ACE treatment for the indicated time, all cells were digested with trypsin, washed once with PBS, and collected. For the apoptosis assay, the cells were resuspended in binding buffer and incubated with Annexin V-FITC and propidium iodide for 15 min at room temperature. For the cell cycle assays, the Cell Cycle and Apoptosis Analysis Kit was used, and the cells were resuspended, fixed with cold 70% ethanol overnight, washed twice with PBS, and then incubated with propidium iodide for 30 min at 37°C. To detect the intracellular ROS levels, cells were stained with DCFH-DA at 37 °C for 20 min. All of the above experiments were performed via flow cytometry with specific signal detectors.

#### Western blot analysis of the cell cycle

To extract total cellular proteins, the cells were resuspended in NP-40 lysis buffer (Beyotime, China) supplemented with PMSF (Beyotime, China) and a protease inhibitor cocktail (Selleck, USA) and placed on ice for 15 min. A BCA protein assay kit (Beyotime, China) was used to quantify the protein concentration, and 15-30 μg of each sample was separated on 8%-12% SDS-PAGE gels (Beyotime, China). The proteins were then transferred onto PVDF membranes (Millipore, USA) and incubated with 5% skim milk (Beyotime, China) for 1 h. Next, the membranes were incubated overnight at 4 °C with a specific primary antibody, followed by secondary antibody incubation at room temperature for 1 h. Finally, the immunoreactive bands were visualized via a Bio-Rad imaging system (Bio-Rad, USA).

#### RNA extraction and quantitative real-time PCR analysis

Quantitative real-time PCR was performed as described previously. RNA was extracted from cells via RNA lysis buffer RNAiso Plus (Takara, China), and up to 1 µg of equal-weight RNA was reverse transcribed to cDNA with an RT Reagent Kit with gDNA Eraser (Yeasen; Shanghai, China). Quantitative RT-PCR was performed via a FastStart SYBR Green Master (Roche, Germany) and LightCycler 96 Instrument (Roche). The gene expression levels were finally normalized to the β-actin and analyzed via the 2^−ΔΔCT^ method. The primers used are listed in the Supplementary Table 2.

### Lipid peroxidation

The cells were labeled via incubation with BODIPY 581/591 C11 dye (Thermo Fisher Scientific, USA) or Liperfluo dye (Dojindo, China) for 30 min at 37 °C. After the cells were washed with PBS, they were detected via an Operetta CLS imaging system (PerkinElmer, USA) or a BD Fortessa flow cytometer (BD Biosciences, USA). The fluorescence intensity was quantified via ImageJ software, or the data were analyzed via FlowJo software. Relative lipid peroxidation was quantified by the green fluorescence intensity of Liperfluo or the fluorescence ratio (FITC/PE ratio (oxidation/reduction ratio)) of C11-BODIPY 581/591 (lipid peroxidation).

Additionally, a Lipid Peroxidation MDA Assay Kit (Beyotime, China) was utilized to detect lipid peroxidation in both cells and tissues. In brief, 100 ml of protein supernatant or standard was mixed with 200 ml of assay solution containing thiobarbituric acid (TBA). The mixture was then heated at 100 °C for 15 min and cooled to room temperature. After centrifugation, the absorbance at 532 nm was measured via a Cytation 5 spectrophotometer. The concentration of MDA was calculated from the standard curve.

#### Tumor organoid models

Tumor organoid models were generated as previously described. Fifty organoids were added to 48-well plates and grown to ~70–100 μm. The ACE concentrations used were 0, 0.01, 0.1, 1, 2, 5, 10, 25, and 50 μM, and triplicate wells were used for each drug concentration. Fresh media containing ACE were changed every 2 days, and the experiments were performed for a total of 6 days. The organoids were then stained with calcein-AM and propidium iodide (15 min at room temperature). Images were captured with a microscope, and the area of live organoids (indicated by green fluorescence) was counted using ImageJ. The organoid size (%) was calculated as follows: sample (total area of living organoids on day 6/total area of living organoids on day 0)/control (total area of living organoids on day 6/total area of living organoids on day 0) × 100.

#### GSH assay

The cells were incubated with ACE, erastin, and RSL3 for 24 h. Following the manufacturer’s instructions (Beyotime, China), the cells were resuspended in Protein Removal Reagent M, and the supernatants were extracted through freeze-thawing with liquid nitrogen. DTNB and glutathione reductase were added to the samples and incubated at room temperature for 5 min. Subsequently, 50 μL of NADPH solution was added, and the change in absorbance at 412 nm was detected with a Cytation 5 spectrophotometer within 25 min. The concentration of GSH was then calculated from the standard curve.

### Cellular Fe^2+^ measurement

The cellular Fe^2+^ level was measured with a FerroOrange probe (Dojindo, China) according to the manufacturer’s instructions. The cells were washed with PBS, 100 μL of PBS containing 1 μM FerroOrange probe was added, and the cells were incubated in the dark for 30 min. The cells were immediately analyzed by flow cytometry or Cytation 5 (TECAN, iControl software). In addition, we determined intracellular Fe^2+^ levels after ACE treatment (1, 2, 5 μM) using Ferrous Iron Colorimetric Assay Kit according to the manufacturer's instructions.

#### Wound-healing assay

CT26 or LoVo (9 × 10^6^) cells were seeded in a 12-well plate to generate a confluent cell monolayer. Gaps were manufactured with a sterile 10 μL pipette tip and then incubated with low-serum medium containing ACE for up to 18 h. Tumor cell migration was evaluated via an inverted microscope.

#### Immunofluorescence microscopy assay

RKO or HCT116 cells were incubated with the indicated treatments before permeabilization with 4% paraformaldehyde and 0.3% Triton X-100. After rinsing with PBS, samples were incubated with 3% BSA at 25 °C for 1 h. Samples were then incubated overnight at 4 °C with the indicated antibodies. The next day, the samples were washed and incubated with secondary antibodies for 1 h at room temperature. Prior to imaging, the nuclei were stained with an anti-fluorescence quencher containing DAPI with or without the FerroOrange probe.

#### Coimmunoprecipitation (IP) assay

Cells were lysed with IP lysis buffer containing protease inhibitors. Lysates were incubated overnight with 2 μg of rat IgG or mouse GPX4 antibody. After 4 h of incubation with agar beads, the beads were rinsed five times with IP lysis buffer and immunoprecipitated by boiling for 10 min in sample buffer.

#### DARTS/MS proteomics analysis

DARTS/MS proteomics analysis was performed according to our previous methods. ACE (100 mM) or DMSO was added to the cell lysate and mixed at 60 rpm for 1 h at room temperature. The samples were subsequently digested with a 1:100–1:500 ratio of enzymes for 30 min at room temperature, after which the reaction was terminated for Western blot analysis. Samples with a 1:300 ratio were selected for LC-MS/MS analysis for proteomic analysis.

#### Quantification and statistical analysis

All experimental data are presented as the mean ± SEM. For differences between two groups, statistical significance was assessed via an unpaired two-tailed t test; one-way ANOVA was used for comparing multiple means; and two-way ANOVA was used for comparing multiple means across conditions. Statistical analysis was performed via GraphPad Prism 9 software. *P* < 0.05 was considered to indicate statistical significance. *P < 0.05, **P < 0.01, ***P < 0.001, ****P < 0.0001.

## Supplementary information

Supplementary information

## Data Availability

We state that the data supporting the results of this study are available from this manuscript and its supplementary information files. The mass spectrometry proteomics data (Figs. 2, 5 and Supplementary Fig. 2) have been deposited to the ProteomeXchange Consortium (https://proteomecentral.proteomexchange.org) via the iProX partner repository^52^ with the dataset identifier PXD064773. The RNA sequencing data (Fig. 2 and Supplementary Fig. 2) discussed in this publication can be accessed at http://itcm.biotcm.net/download.html.^53^
