# Supplementary material for: Acevaltrate as a novel ferroptosis inducer with dual targets of PCBP1/2 and GPX4 in colorectal cancer
Source: Signal Transduct Target Ther. 2025 Jul 7;10:211. doi: 10.1038/s41392-025-02296-7 (PMC12230175; doi:10.1038/s41392-025-02296-7)
Supplement: Supplementary file 1 — Supplementary information [file 41392_2025_2296_MOESM1_ESM.pdf]

# Supplementary Materials for

# Acevaltrate as a novel ferroptosis inducer with dual targets of PCBP1/2 and GPX4 in colorectal cancer

Dianping Yu[1†], Hongmei Hu[1,2†], Qing Zhang[1†], Chengji Wang[3†], Mengting Xu[1], Hanchen Xu[2], Xiangxin Geng[1], Minchen Cai[1], Hongwei Zhang[1], Mengmeng Guo[1], Dong Lu[1], Hanchi Xu[1], Linyang Li[1], Xing Zhang[1], Ruling Shen[2], Sheng Lin[4*], Qun Wang[1*], Weidong Zhang[1,5,6*], Sanhong Liu[1*]

[†] Authors contributed equally to this work

Corresponding to: Sanhong Liu (liush@shutcm.edu.cn), Weidong Zhang (wdzhangy@hotmail.com), Qun Wang (qunwang0523@163.com), Sheng Lin ( lsznn@bucm.edu.cn)

**This PDF file includes:**

Figures S1 to S15
Tables S1 to S2

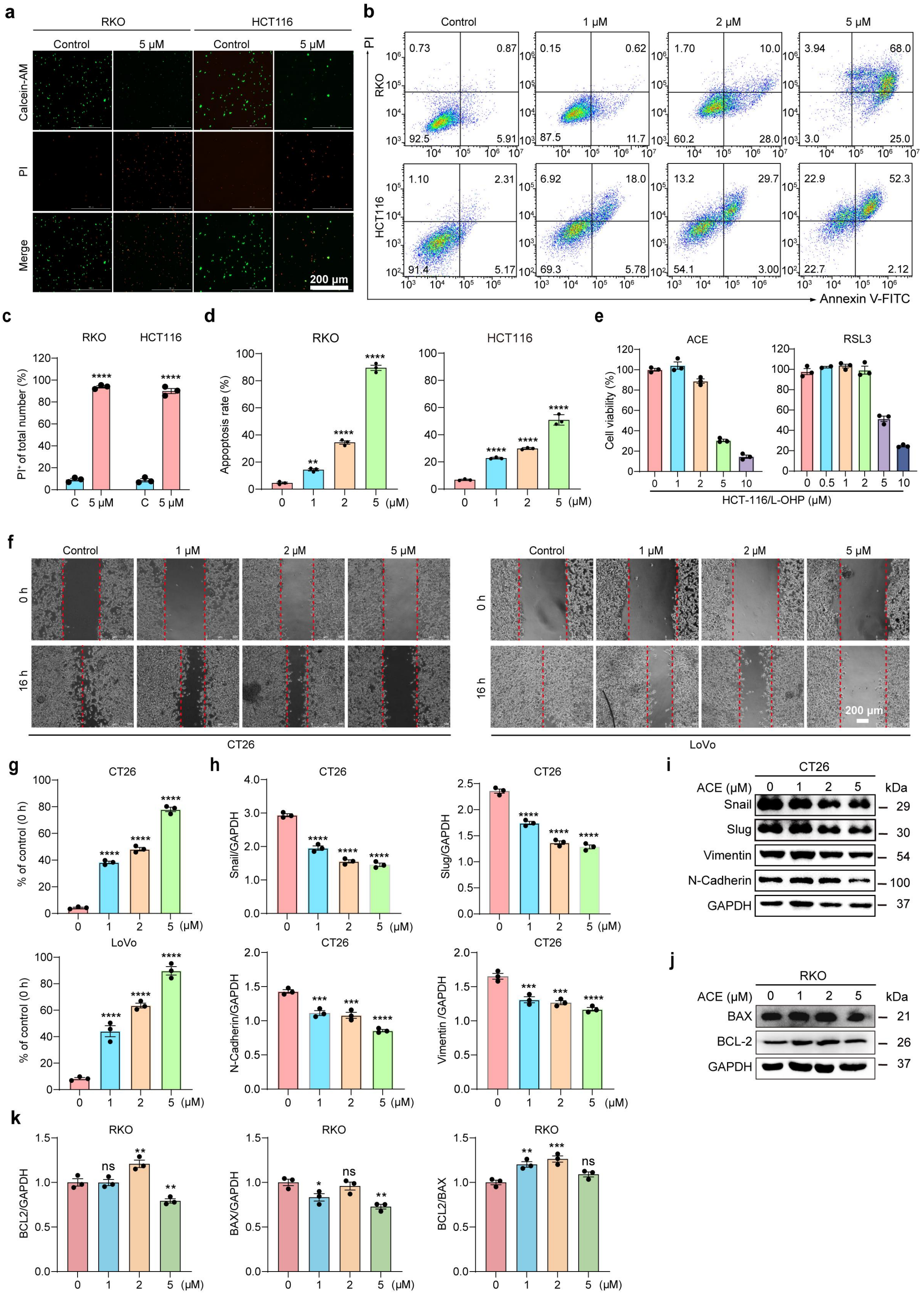


**Supplementary Fig. 1 ACE inhibits cell migration and drug resistance. a, b** Cell death analysis of RKO and HCT116 cells treated with ACE (1, 2, 5 μM) for 24 hours using calcein/PI staining **(a)** and flow cytometry **(b)**. Scale bars, 200 μm. **c** Quantification of calcein/PI-stained RKO and HCT116 cells showing cell death after

ACE treatment. **d** Quantitative analysis of **(b)**. (n = 3, error bars represent SEM, one-way ANOVA). **e** CCK-8 analysis of the inhibition ratio in HCT116/L-OHP cells treated with oxaliplatin and ACE. **f, g** Cell migration analysis of CT26 and LOVO cells treated with ACE (1, 2, 5 μM) for 16 hours using wound healing assays and quantitative analysis are shown in **(g)**. (n = 3, error bars represent SEM, one-way ANOVA). **h, i** Western blot analysis of Snail, Slug, Vimentin, and N-cadherin after ACE treatment for 16 h **(i)** and quantification **(h)**. **j, k** Western blot analysis of BCL2 and BAX protein levels in RKO cells after treatment with ACE, which are proteins associated with apoptosis; **k** quantification of **(j)**. n = 3, data are shown as mean ± SEM. The experiments consisted of three biological replicates with similar results. One-way ANOVA was performed in **(d, g, h and k)**; Two-tailed unpaired Student's t-test was performed in **(c)**. $*P < 0.05$, $**P < 0.01$, $***P < 0.001$, $****P < 0.0001$; ns not significant.

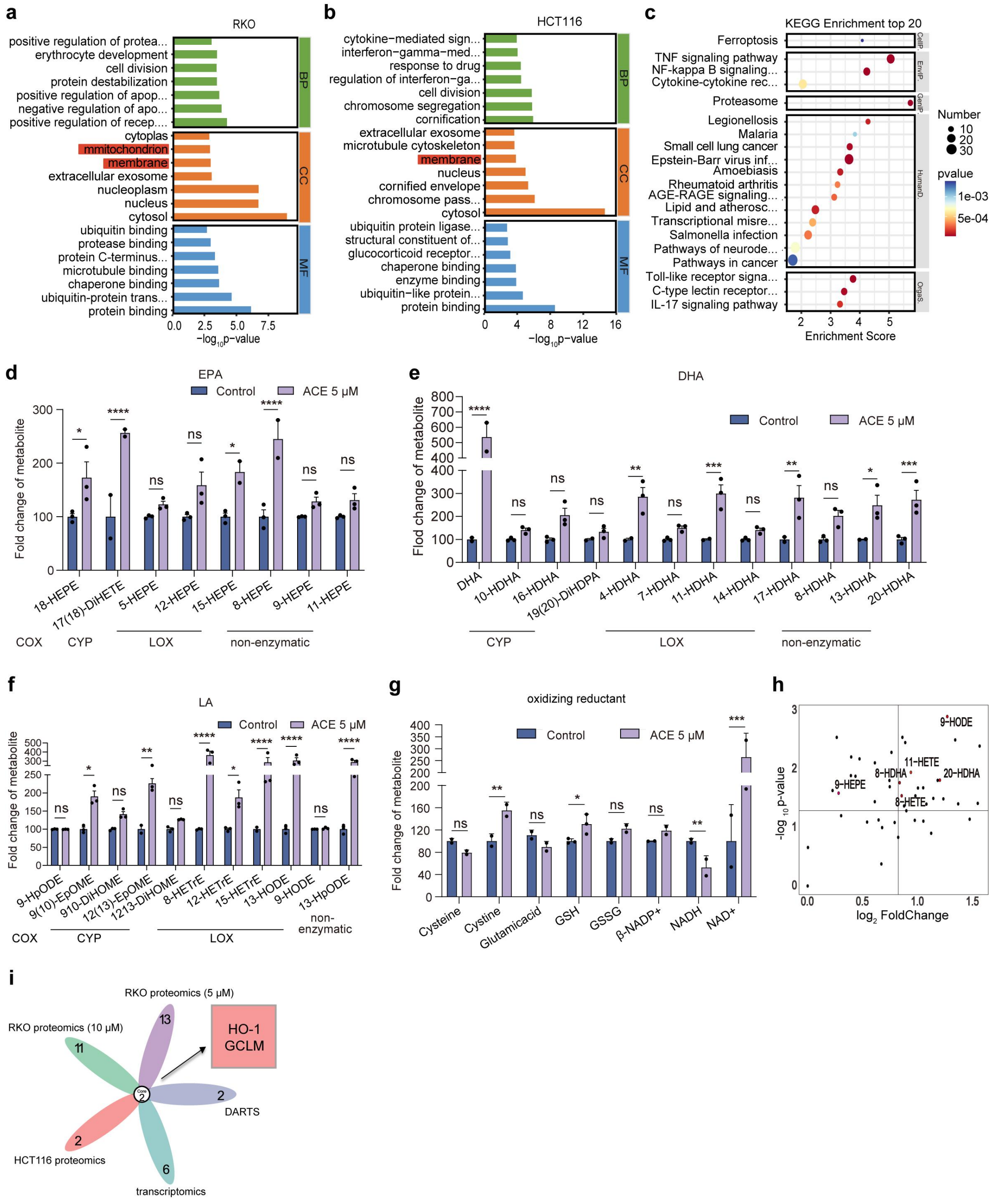


**Supplementary Fig. 2 Ferroptosis is predicted to be a potential pathway for ACE based on a multiomics strategy. a, b** GO analysis of differentially expressed proteins in RKO **(a)** and HCT116 **(b)** cells. **c** Potential pathway analysis by KEGG enrichment based on differentially expressed mRNAs in MCF7 cells via transcriptomics. **d-f** EPA **(d)**, DHA **(e)** and LA **(f)**, and metabolite levels in RKO cells treated with ACE (5 μM) compared with DMSO. **g** Analysis of the amount of the oxidizing reductant in ACE-treated RKO cells. **h** Scatterplot of oxidized polyunsaturated fatty acids, in

which nonenzymatic metabolites are labeled. **i** Flower plot showing the ferroptosis core genes of the differentially expressed proteins after ACE treatment. The experiments consisted of three biological replicates with similar results. Two-tailed unpaired Student's t-test was performed in **(d-g)**. *$P < 0.05$, **$P < 0.01$, ***$P < 0.001$, ****$P < 0.0001$; ns not significant.

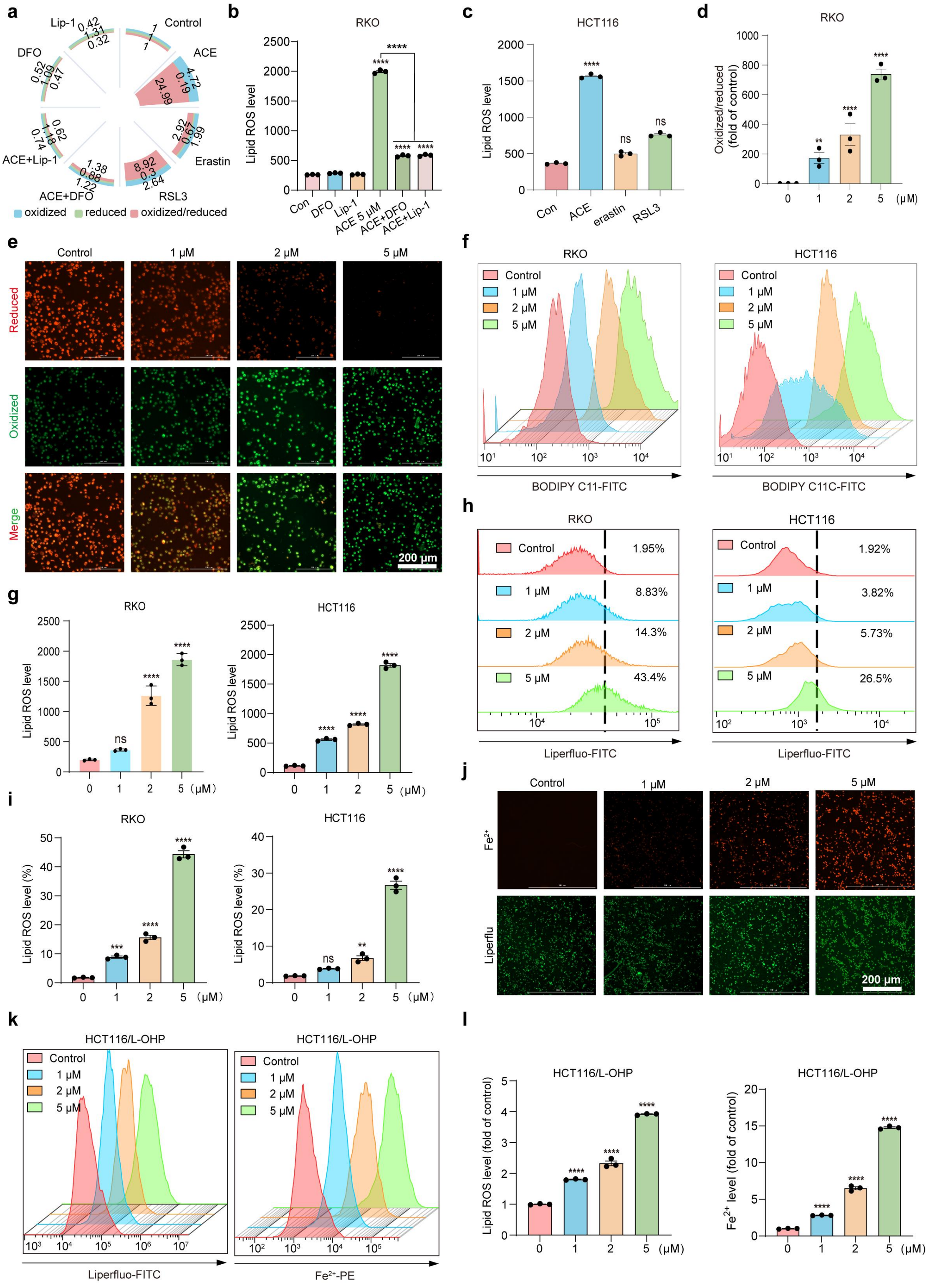


**Supplementary Fig. 3 ACE induces lipid peroxidation accumulation and ferroptosis in colorectal cancer cells. a-c** Statistics of the microscopy images **(a)** and flow cytometry **(b, c)** showing the lipid peroxidation levels in cells stained with the BODIPY-C11 probe after the indicated treatments, related to **Fig. 3a, b**. **d, e**

Dose-dependent lipid peroxidation accumulation of ACE in RKO cell lines **(e)** and quantitative analysis are shown in **(d)**. **f, g** Flow cytometry analysis **(f)** and statistical analysis **(g)** of lipid peroxidation levels in ACE-treated RKO and HCT116 cells using the BODIPY-C11 probe. **h, i** Liperfluo staining showing lipid peroxidation levels in RKO and HCT116 cells after ACE treatment for 3 h. Statistical analysis of the flow cytometry data is shown in **(i)**. **j-l** HCT116/L-OHP cells were treated with ACE for the indicated times, and lipid peroxidation and $Fe^{2+}$ levels were measured using fluorescence staining **(j)** or flow cytometry **(k)**. The quantitative results are shown in **(l)**. Data are shown as mean ± SEM. n = 3 biological replicates with similar results. All the statistical analyses were performed via one-way ANOVA. $^{**}P < 0.01$, $^{***}P < 0.001$, $^{****}P < 0.0001$; ns not significant.

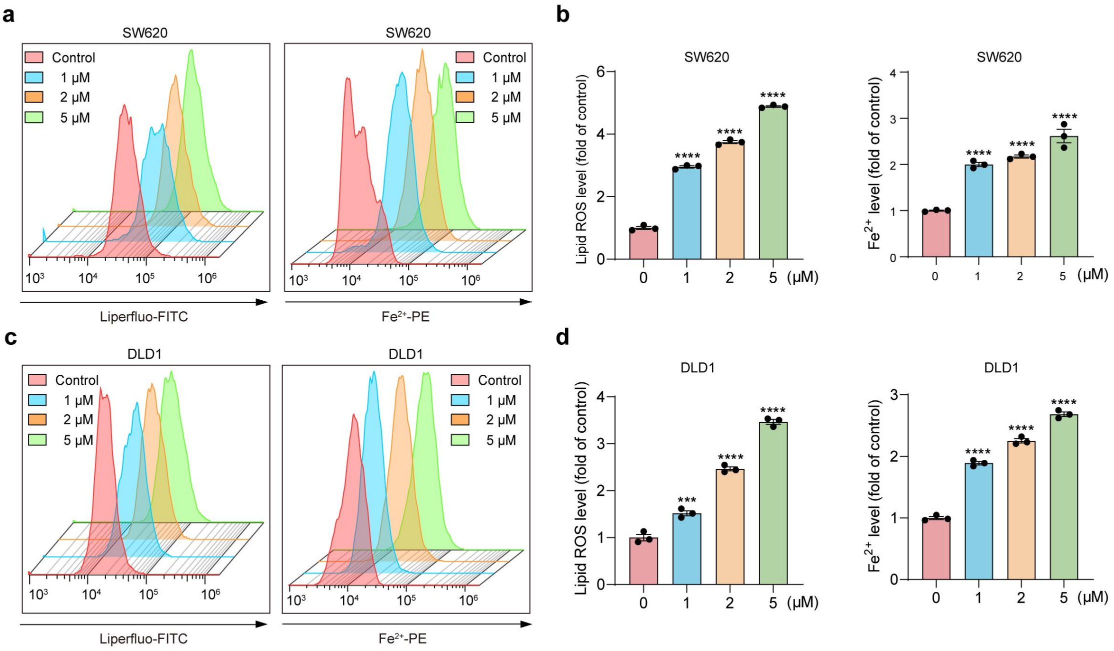


**Supplementary Fig. 4 ACE significantly elevated lipid peroxidation and $Fe^{2+}$ levels in colorectal cancer. a-d** Flow cytometry showing SW620 **(a)** and DLD1 **(c)** cells were treated with ACE for the indicated times. The quantitative results are shown in **(b, d)**. Data are shown as mean ± SEM. n = 3 biological replicates with similar results. All the statistical analyses were performed via one-way ANOVA.

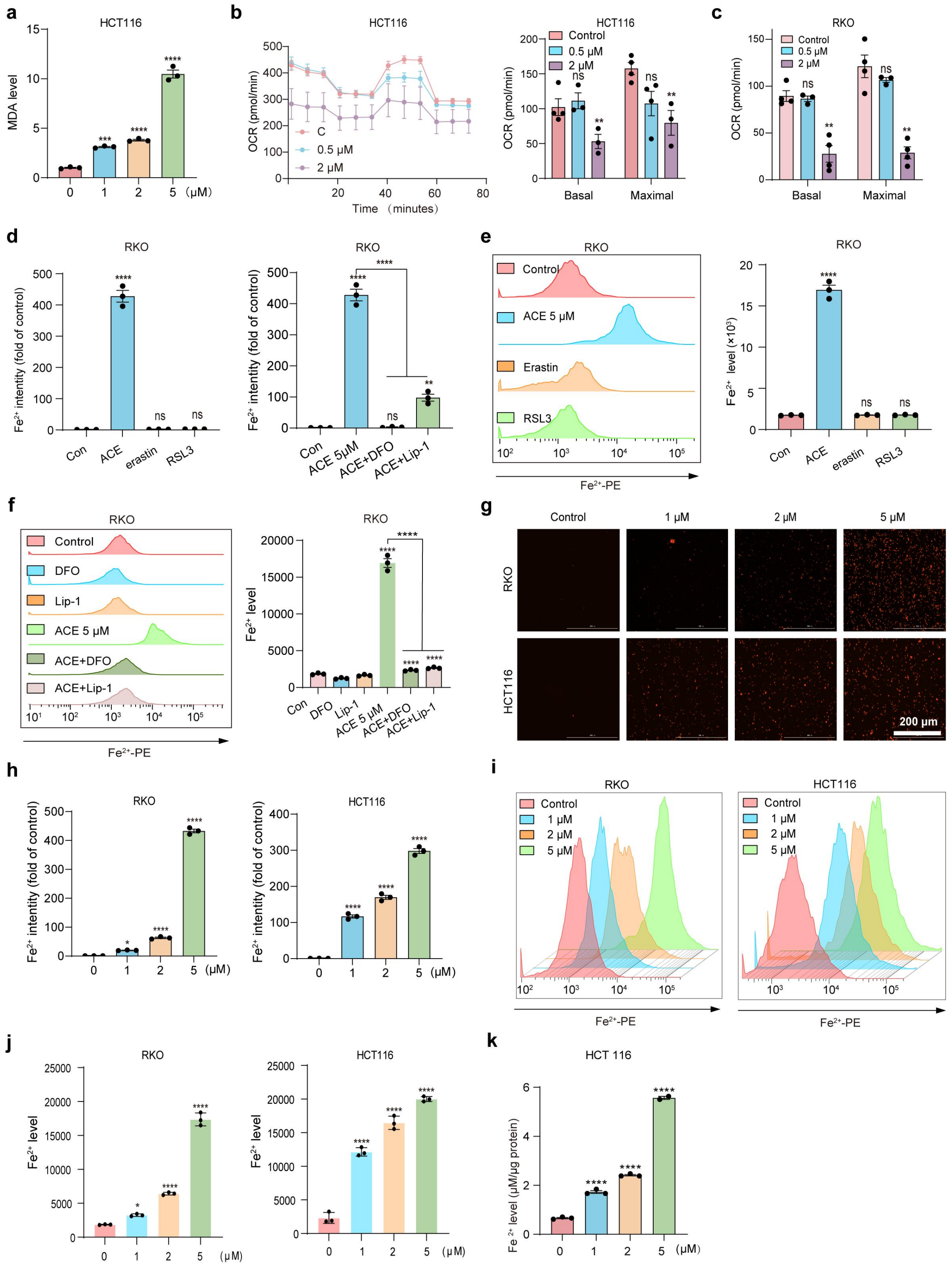


**Supplementary Fig. 5 ACE induces ferroptosis via increasing of $Fe^{2+}$ levels in colorectal cancer cells. a** Statistics of the malondialdehyde (MDA) assay showing the lipid peroxidation levels of HCT116 cells treated with ACE. **b** Mitochondrial respiration analysis and statistical analysis of HCT116 cells treated with ACE (0.5 and 2 μM, 12 h) or DMSO (Ctrl). **c** Statistical analysis of mitochondrial respiration

analysis related to **Fig. 3f**. **d** Statistics of $Fe^{2+}$ levels under the indicated treatments, related to **Fig. 3g. e, f** Analysis of $Fe^{2+}$ levels and quantification in ACE-treated RKO cells compared with ferroptosis inducer erastin or RSL3 **(e)** or in the presence or absence of DFO and Lip-1 **(f)**. **g-j** Dose-dependent accumulation of $Fe^{2+}$ in ACE in RKO and HCT116 cells as determined by microscopy **(g)** or flow cytometry **(i)** after FerroOrange staining. Quantitative analysis is shown in **(h)** and **(j)**, respectively. **k** ferrous ion colorimetric assay analysis of $Fe^{2+}$ level in RKO cells treated with ACE. Data are shown as mean ± SEM. n = 3 biological replicates with similar results. All statistical analyses except **(b, c)** performed were performed by one-way ANOVA; Two-way ANOVA was performed in **(b, c)**. $*P < 0.05$, $**P < 0.01$, $****P < 0.0001$; ns not significant.

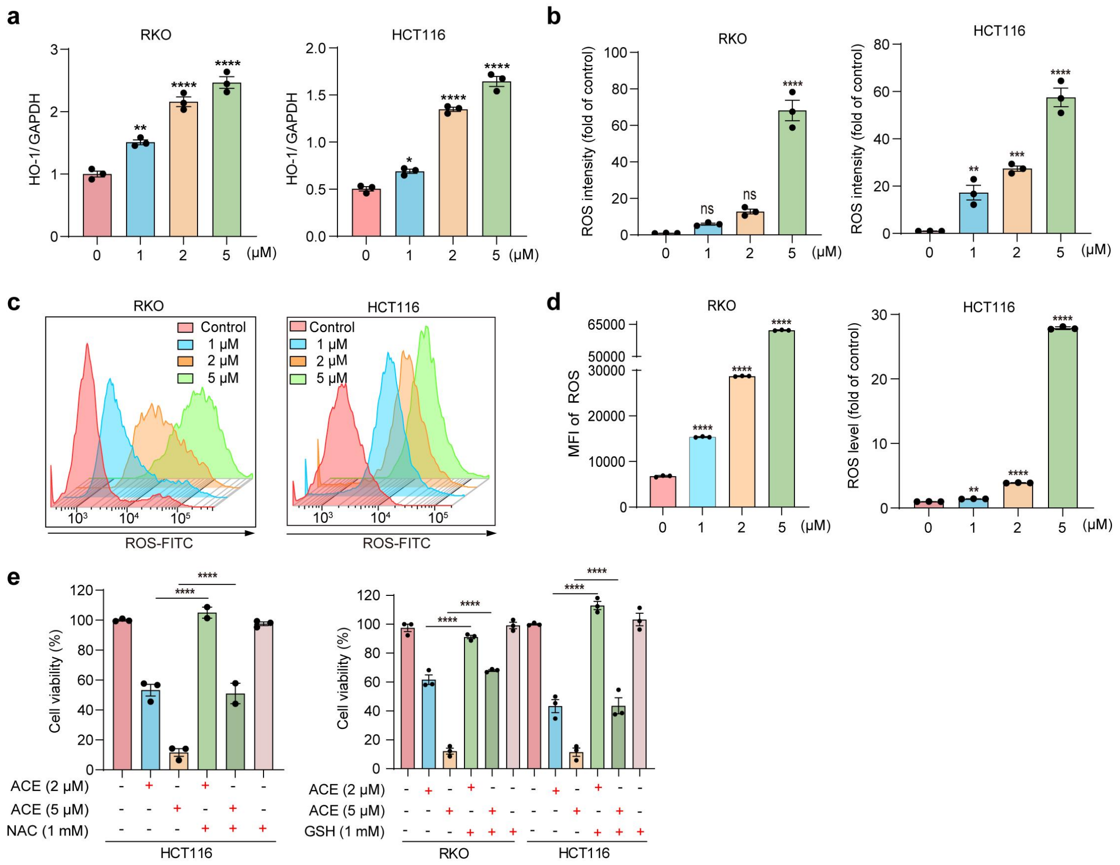


**Supplementary Fig. 6 ACE elevates ROS levels and induces ferroptosis in colorectal cancer cells. a** Statistical analysis of HO-1 protein levels in RKO and HCT116 cells after treatment with various concentrations of ACE. **b** Statistics of ROS levels under the indicated treatments (related to **Fig. 3h**). **c, d** ROS level analysis **(c)**

and statistical analysis **(d)** of RKO and HCT116 cells treated with ACE. **e** Cell viability analysis of RKO and HCT116 cells after treatment with ACE with or without the ROS scavenger NAC or GSH. $^{**}P < 0.01$, $^{***}P < 0.001$, $^{****}P < 0.0001$; ns not significant.

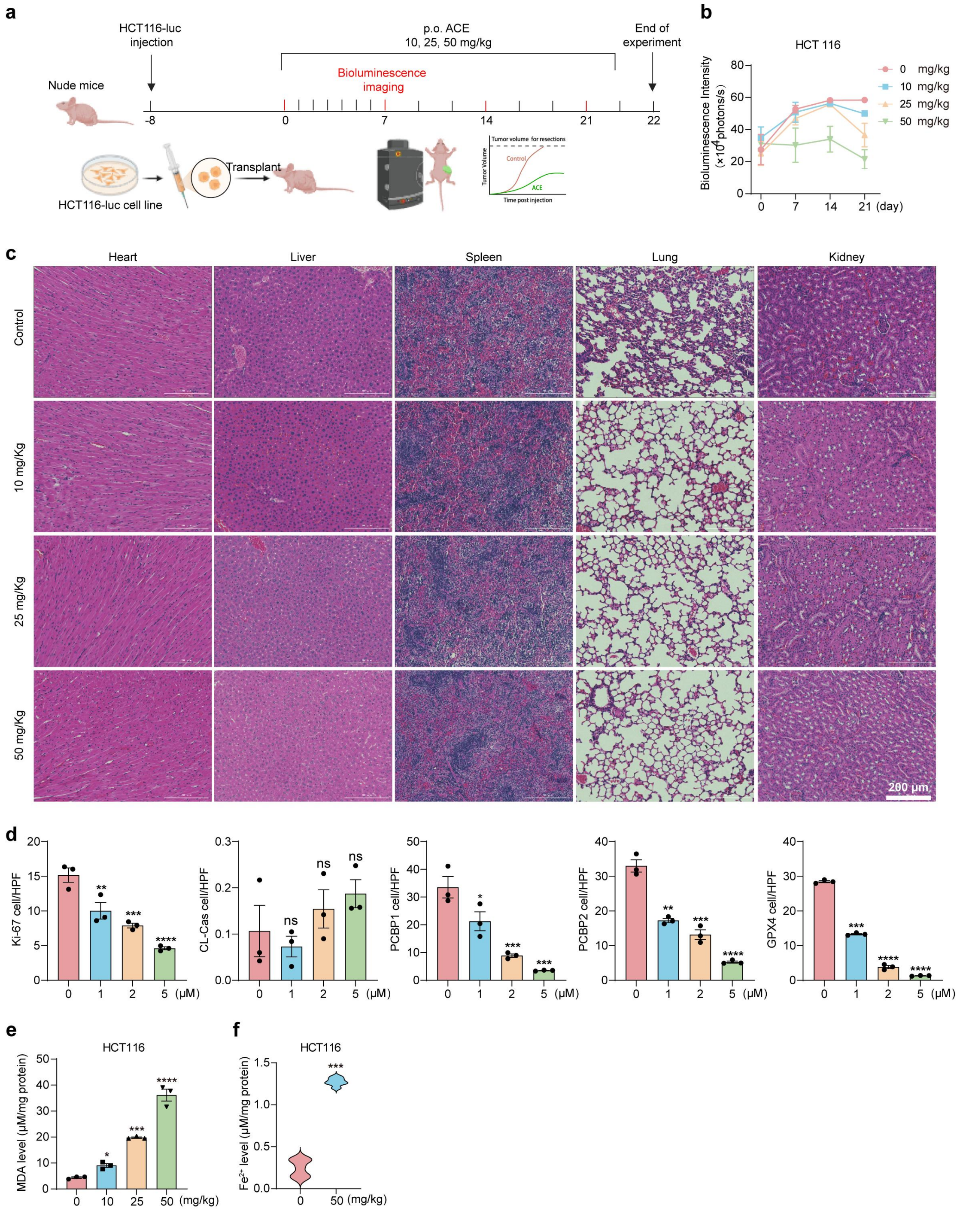


**Supplementary Fig. 7 ACE suppresses tumor growth in mice through ferroptosis.**
**a** HCT116-luc cells were injected into BALB/c-nu mice on day -8, ACE was

administered orally daily from day 0, and bioluminescence imaging was performed on days 0, 7, 14, and 21. **b** Statistics of the bioluminescence intensity of HCT116-luc tumors under ACE treatment. **c** Representative HE staining results for the heart, lung, liver, spleen, and kidney in corn oil- or ACE-treated BALB/c-nu mice. **d** Quantification of the IHC staining results is shown related to **Fig. 4l**. **e, f** Quantification of the MDA **(e)** and $Fe^{2+}$ **(f)** levels in the tumors of HCT116-luc mice after ACE treatment. Data are shown as mean ± SEM. The experiments consisted of three biological replicates with similar results. Two-tailed unpaired Student's t test was performed in **(f)**; One-way ANOVA was performed in **(d and e).** Two-way ANOVA was performed in **(b)**. *$P < 0.05$, **$P < 0.01$, ***$P < 0.001$, ****$P < 0.0001$; ns not significant.

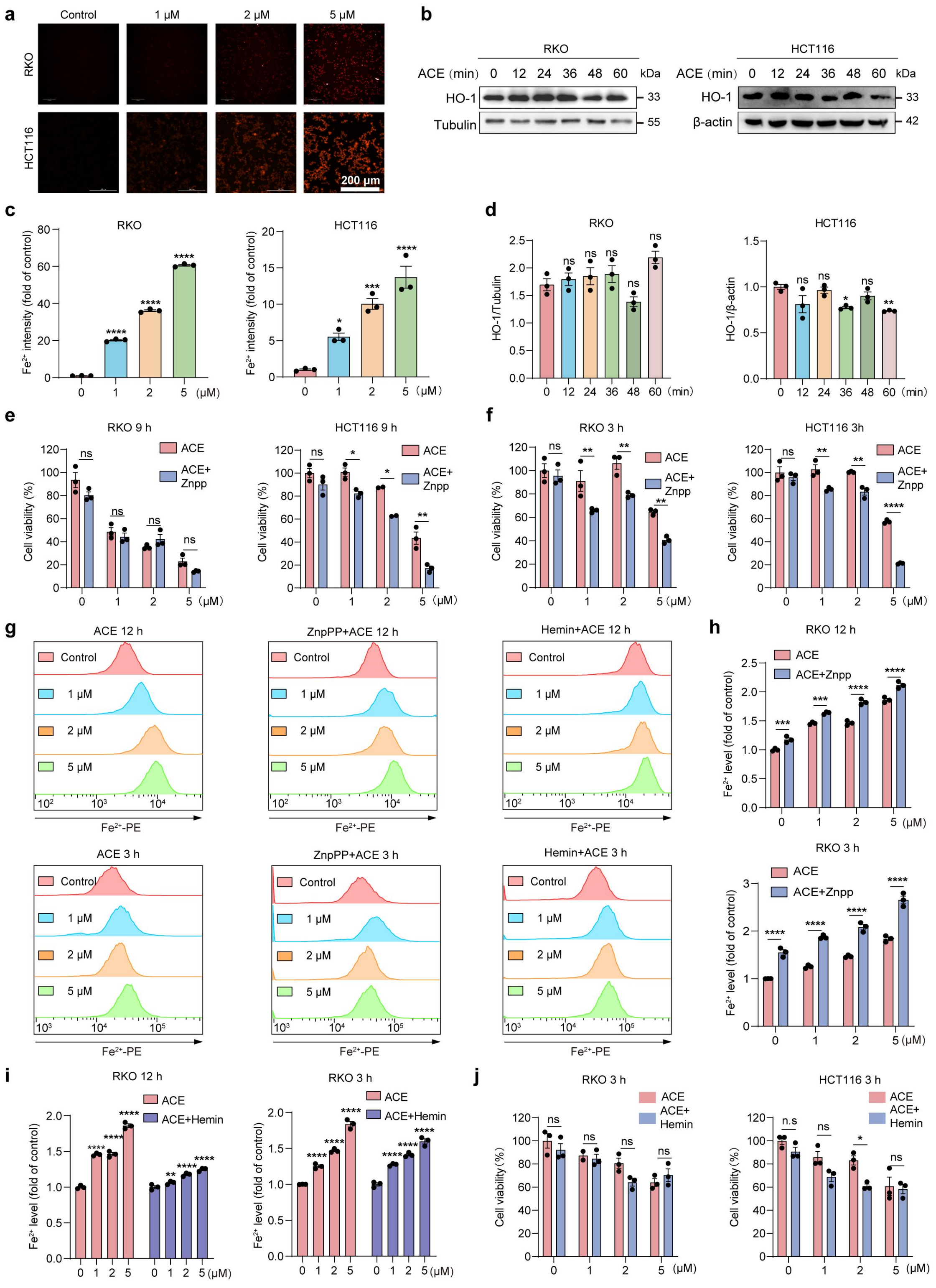


**Supplementary Fig. 8 ACE increases $Fe^{2+}$ levels independent of HO-1. a,c** FerroOrange staining **(a)** and statistics **(c)** showing $Fe^{2+}$ levels in RKO and HCT116 cells treated with ACE for 12 min. **b, d** Protein levels **(b)** and statistics **(d)** of HO-1 expression in RKO and HCT116 cells treated with ACE for 1 h determined via Western blot. **e, f** RKO and HCT116 cells were treated with ACE in the presence or

absence of the HO-1 inhibitor Znpp for 9 h **(e)** or 3 h **(f)**, and cell viability was determined using a CCK-8 assay. **g-i** Flow cytometry **(g)** and statistics **(h, i)** showing $Fe^{2+}$ levels in RKO cells treated with different concentrations of ACE in the presence or absence of Znpp **(h)** or hemin **(i)** for 12 h or 3 h. **j** Cell viability analysis of RKO and HCT116 cells treated with ACE with or without the HO-1 agonist Hemin for 3 h. Data are shown as mean ± SEM. The experiments consisted of three biological replicates with similar results. One-way ANOVA was performed in **(c, d)**; Two-way ANOVA was performed in **(e, f, and h-j)**. *$P$ < 0.05, **$P$ < 0.01, ***$P$ < 0.001, ****$P$ < 0.0001; ns not significant.

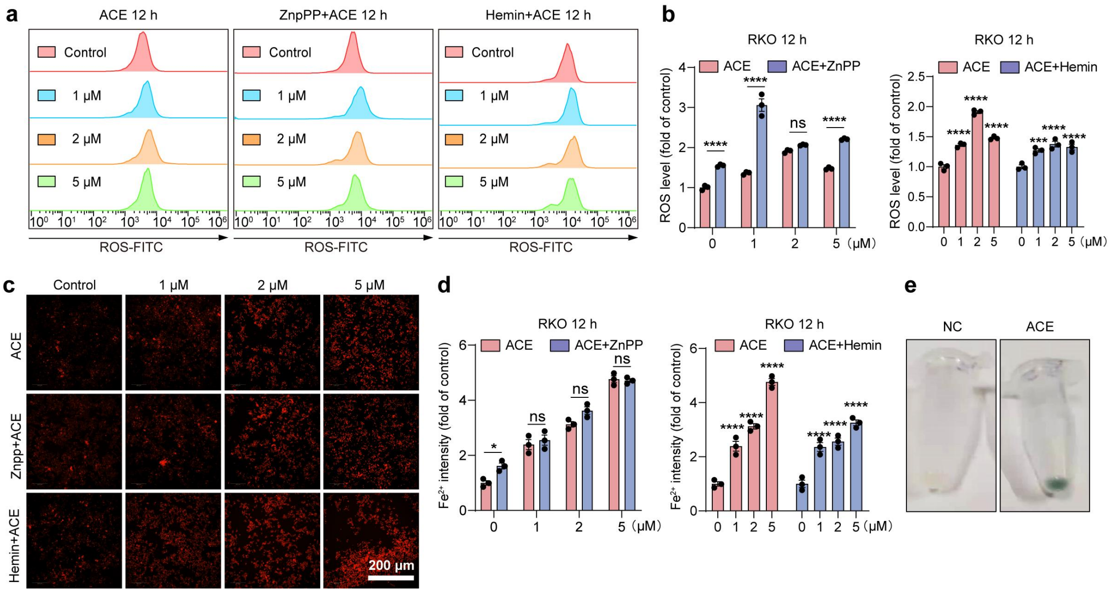


**Supplementary Fig. 9 ACE induces ferroptosis independent of HO-1. a. b** Flow cytometry analysis **(a)** of ROS levels and quantification **(b)** in ACE-treated RKO cells for 12 h. **c, d** FerroOrange staining showing $Fe^{2+}$ levels in ACE-treated RKO cells in the presence or absence of Znpp or Hemin for 12 h. Quantitative results are shown in **(d)**. **e** Appearance of cells after ACE (5 μM) treatment for 12 h. Data are shown as mean ± SEM. n = 3 biological replicates with similar results. All the statistical analysis were performed via two-way ANOVA. *$P$ < 0.05, ***$P$ < 0.001, ****$P$ < 0.0001; ns not significant.

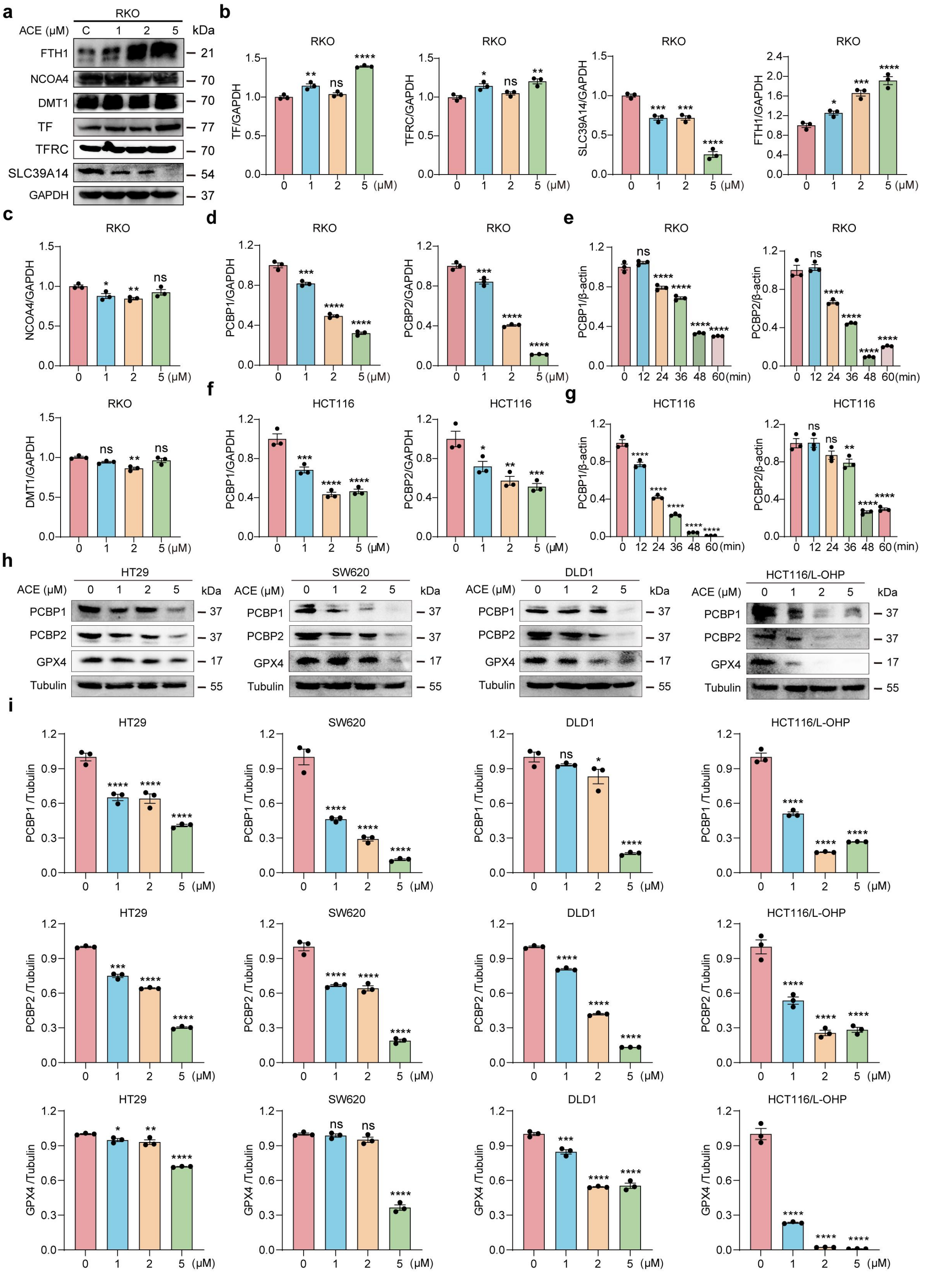


**Supplementary Fig. 10 ACE downregulates PCBP1/2 in colorectal cancer cells.** **a-c** Western blot analysis **(a)** and statistics **(b, c)** of FTH1, NCOA4, TF, TFRC, DMT1, and SLC39A14 protein levels in RKO cells after treatment with ACE, which are proteins associated with iron metabolism based on proteomic results. **d, e** Statistics of protein levels in RKO cells after treatment with dose-dependent **(d)** or time-dependent

**(e)** ACE, related to **Fig. 5a**. **f, g** Statistics of protein levels in HCT116 cells after treatment with dose-dependent **(f)** or time-dependent **(g)** ACE, related to **Fig. 5b**. **h, i** Western blot analysis of PCBP1, PCBP2, and GPX4 protein levels in HT29, SW620, DLD1 and HCT116/L-OHP cells after treatment with dose-dependent ACE. The quantitative results are shown in **(i)**. **P* < 0.05, ***P* < 0.01, ****P* < 0.001, *****P* < 0.0001; ns not significant.

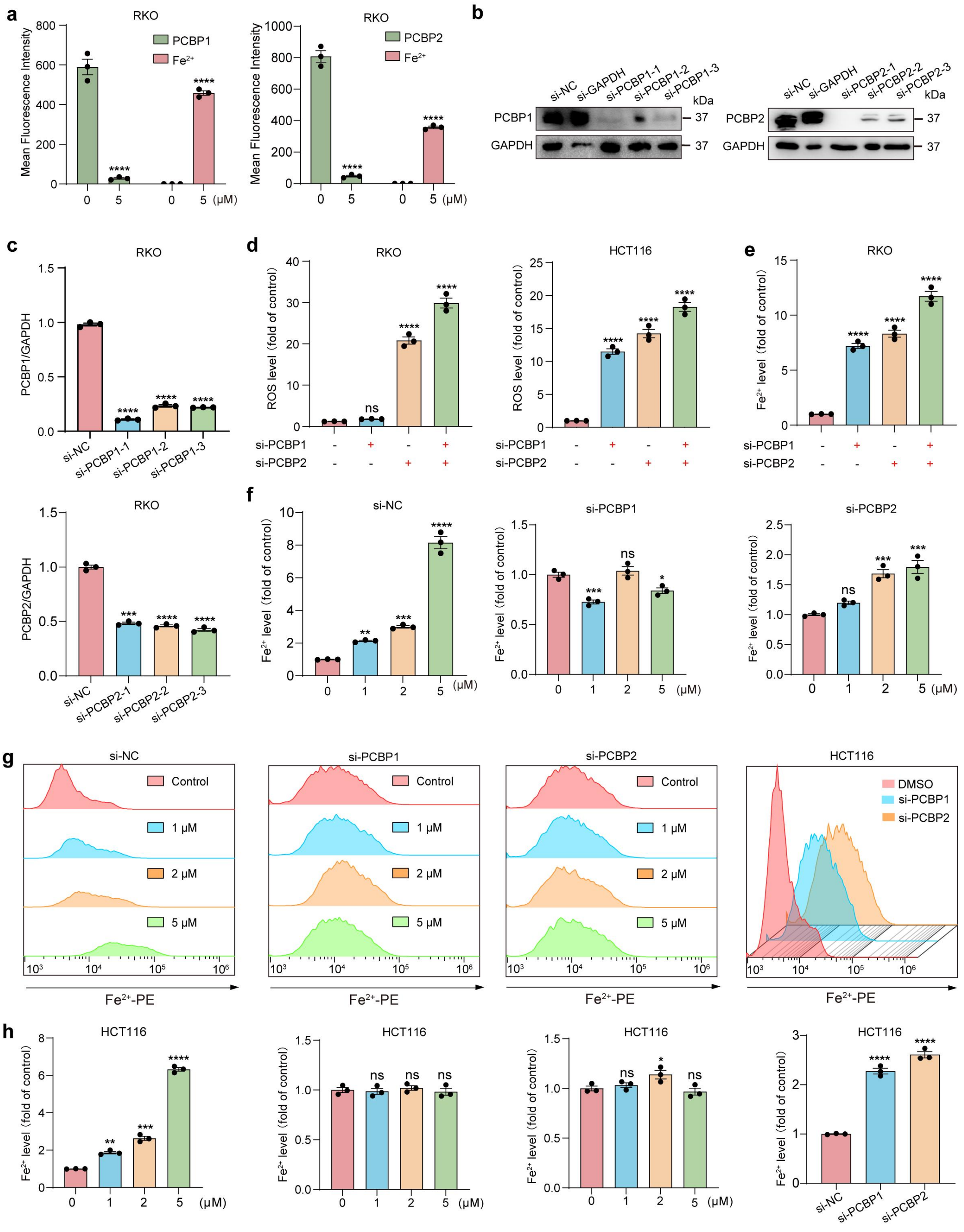

**Supplementary Fig. 11 ACE downregulates PCBP1/2 to increase $Fe^{2+}$ levels. a** Quantification of PCBP1/2 and $Fe^{2+}$ fluorescence intensity, related to **Fig. 5d**. **b, c** Western blot analysis of the knockdown efficiency of PCBP1 and PCBP2 in RKO cells transfected with PCBP1 and PCBP2 siRNAs, and the knockdown efficiency was quantified in **(c)**. **d, e** Statistics of ROS **(d)** and $Fe^{2+}$ **(e)** levels in PCBP1- or PCBP2-knockdown RKO and HCT116 cells, related to **Fig 5e, f**. **f** Statistics of $Fe^{2+}$ levels in ACE-treated RKO cells with or without knockdown of PCBP1 and PCBP2, related to **Fig 5h**. **g, h** Flow cytometry analysis **(g)** and statistics **(h)** of $Fe^{2+}$ levels in ACE-treated PCBP1- or PCBP2-knockdown HCT116 cells. Data are shown as mean ± SEM. n = 3 biological replicates with similar results. All statistical analysis were performed via one-way ANOVA; *$P < 0.05$, **$P < 0.01$, ***$P < 0.001$, ****$P < 0.0001$; ns not significant.

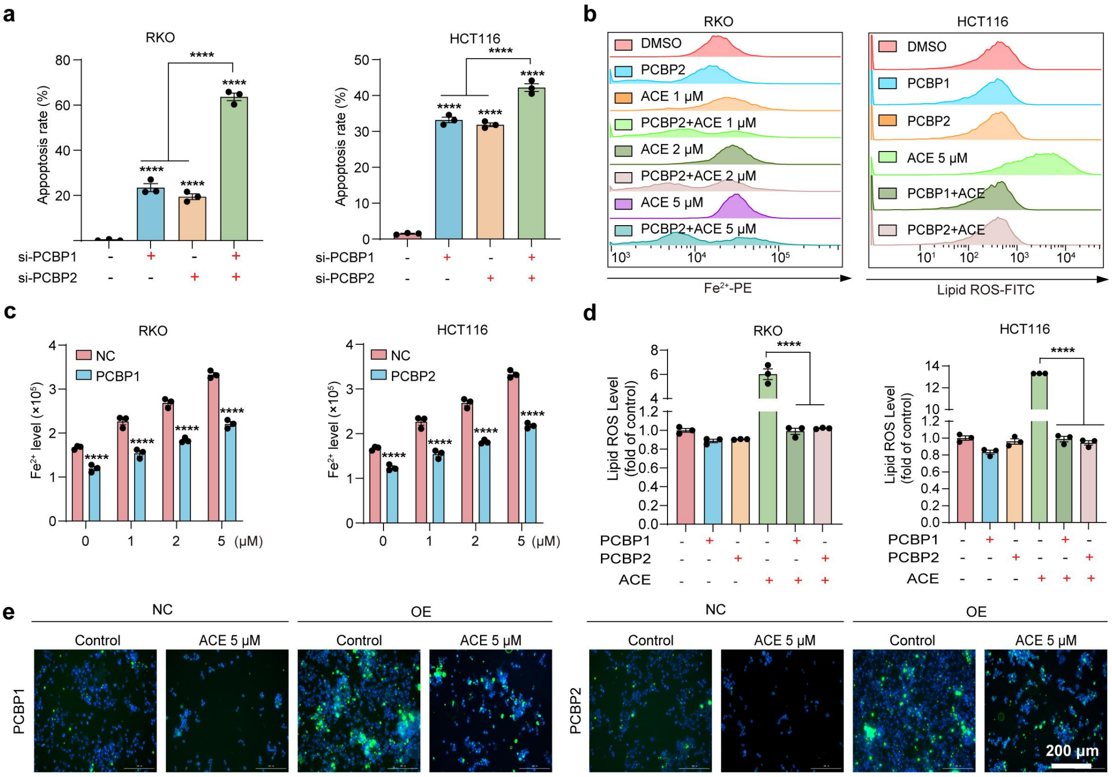


**Supplementary Fig. 12 ACE induces ferroptosis via targeting PCBP1/2. a** Quantification of cell death in RKO and HCT116 cells after transfection with siRNAs targeting PCBP1 and PCBP2 for 48 h. **b** $Fe^{2+}$ levels and lipid ROS levels in PCBP1- or PCBP2-overexpressing cells treated with ACE. **c, d** Statistics of $Fe^{2+}$ **(c)** and lipid

ROS **(d)** levels in PCBP1- or PCBP2-overexpressing RKO and HCT116 cells, related to **Fig. 5l, m** and **Supplementary Fig. 12b**, respectively. **e** Immunofluorescence images of PCBP1/2 after overexpressing PCBP1 or PCBP2 in the presence or absence of ACE. Data are shown as mean ± SEM. The experiments consisted of three biological replicates with similar results. One-way ANOVA was performed in **(d)**; Two-way ANOVA was performed in **(c)**. $*P < 0.05$, $**P < 0.01$, $***P < 0.001$, $****P < 0.0001$; ns not significant.

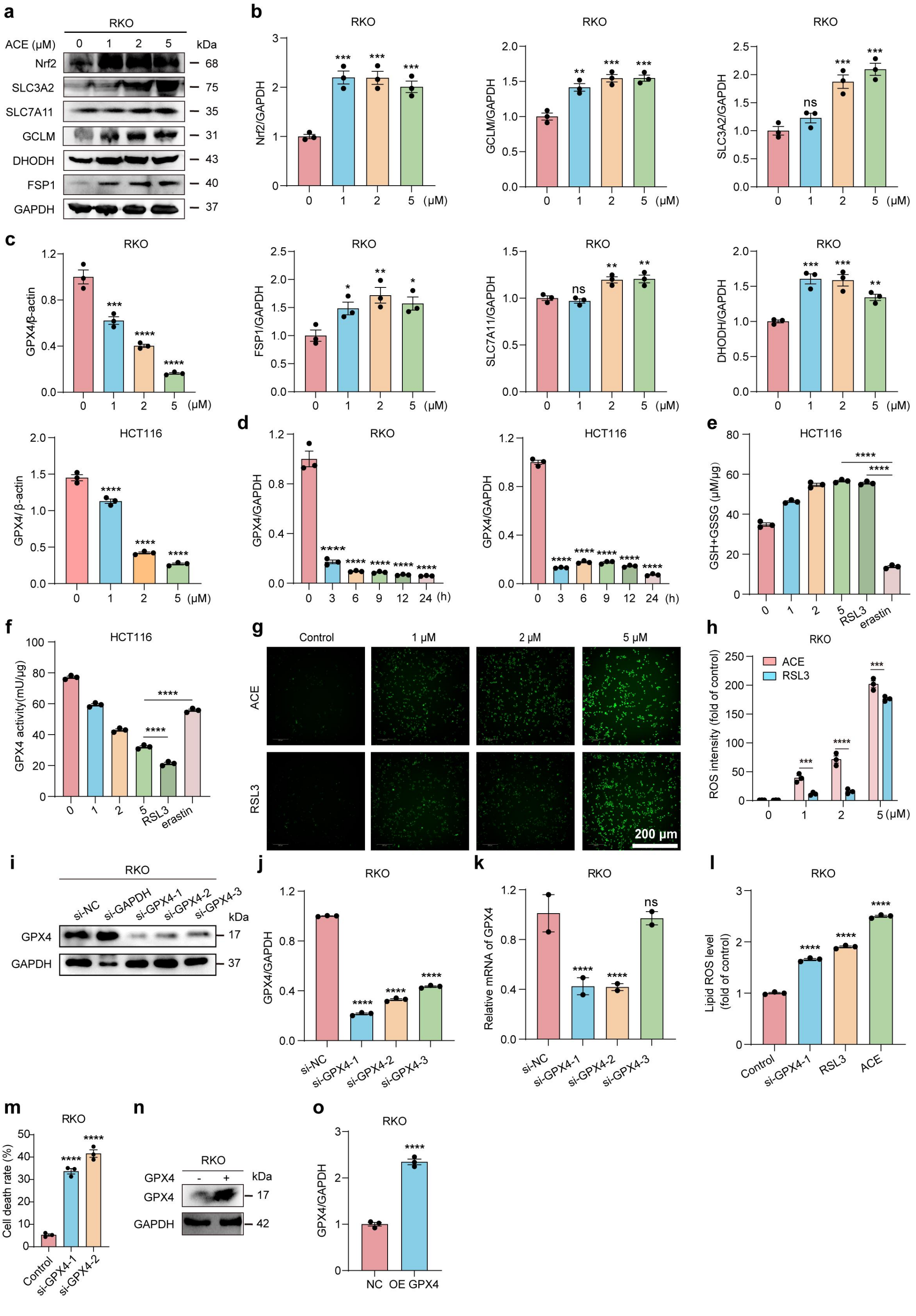


**Supplementary Fig. 13 ACE induces GPX4 depletion. a, b** Western blot analysis of Nrf2, SLC3A2, SLC7A11, GCLM, DHODH, and FSP1 protein levels in RKO cells after treatment with ACE, which are proteins associated with ferroptosis antioxidant

factors based on proteomics and transcriptomics results. **(b)** quantification **(a)**. **c, d** Statistics of GPX4 protein levels in RKO and HCT116 cells after treatment with dose-dependent **(c)** or time-dependent **(d)** ACE. **e, f** Analysis of GSH levels **(e)** and GPX enzyme activity **(f)** in HCT116 cells after treatment with ACE, RSL3, or erastin. **g, h** DCFH-DA staining showing intracellular ROS levels in RKO cells after treatment with ACE or the ferroptosis inducer RSL3. **(h)** quantification **(g)**.(n = 3, scale bars, 200 μm). **i-k** Western blot **(i)** and qPCR **(k)** analysis showing the knockdown efficiency of GPX4 in RKO cells transfected with siRNAs targeting GPX4, and the quantitative results of **(i)** are shown in **(j)**. **l** Statistics of lipid ROS levels in RKO cells treated with the indicated reagents. **m** Quantification of cell death in GPX4-knockdown RKO cells using flow cytometry. **n, o** Overexpression analysis of GPX4 expression after transfection of the GPX4 plasmid into RKO cells for 48 h. Quantitative results are shown in **(o).** Data are shown as mean ± SEM. n = 3 biological replicates with similar results. All the statistical analysis except **(o)** were performed via one-way ANOVA. Two-tailed unpaired Student's t-test was performed in **(o)**. *$P$ < 0.05, **$P$ < 0.01, ***$P$ < 0.001, ****$P$ < 0.0001; ns not significant.

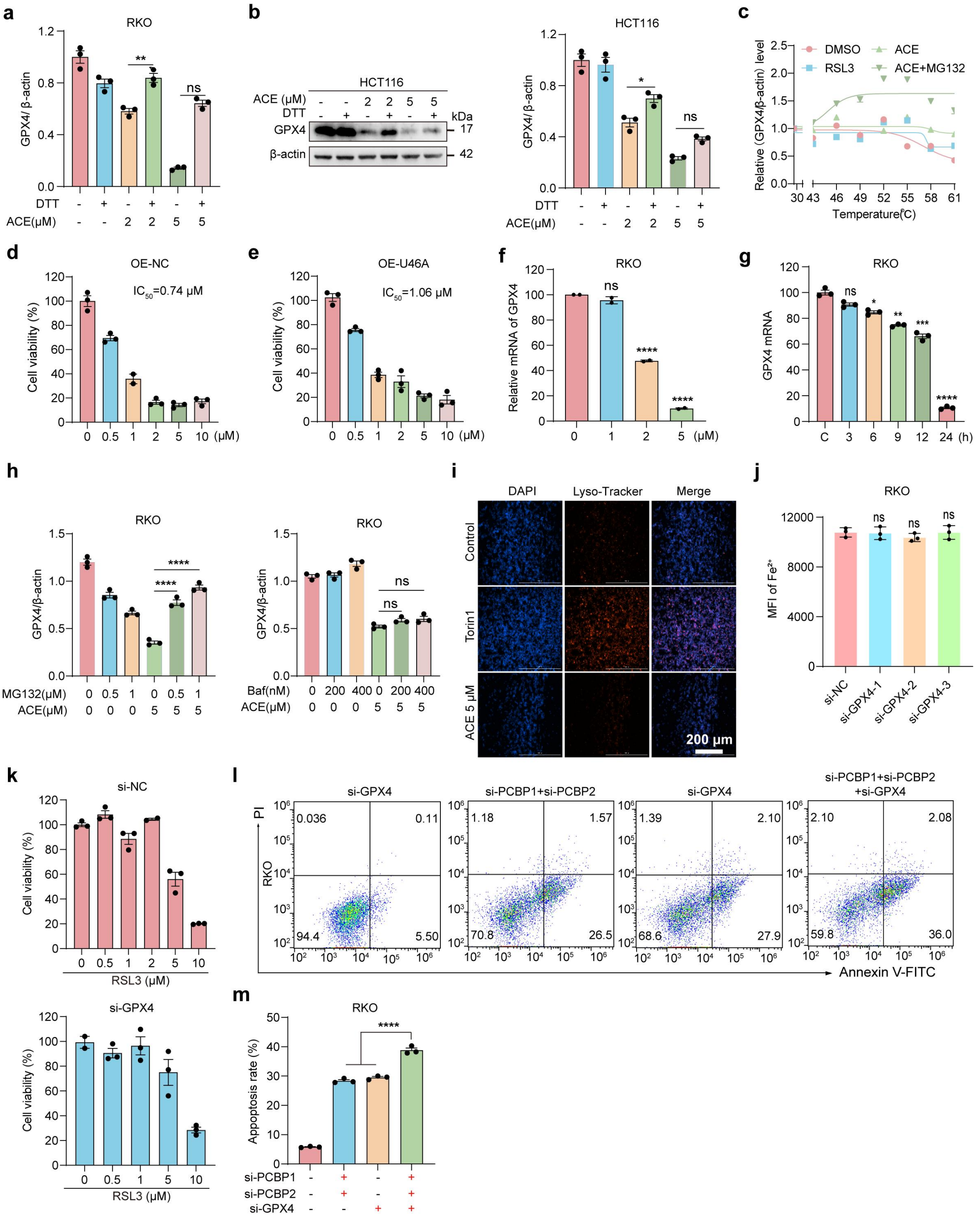

**Supplementary Fig. 14 PCBP1, PCBP2, and GPX4 synergistically promoted ferroptosis in colorectal cancer cells. a-c** Western blot analysis **(b)** and statistics **(a, c)** of GPX4 expression in RKO and HCT116 cells treated with ACE with or without DTT for 24 h (related to **Fig. 7h**). **c** Quantification of GPX4 protein levels in **Fig. 7j**. **d, e** Cell viability analysis showing the dose-dependent toxicity of ACE (0.5, 1, 2, 5, and 10 μM) in OE NC **(d)** and OE **(e)** GPX4-U46A RKO cells using a CCK-8 assay. **f, g** qPCR analysis of GPX4 expression in RKO cells treated with ACE. **h** GPX4 protein

levels in ACE-treated RKO cells in the presence or absence of MG132 or Baf. **i** LysoTracker staining images showing RKO cells treated with ACE or Torin1 for 12 h. **j, k** $Fe^{2+}$ levels **(j)** and cell viability **(k)** analysis of GPX4-knockdown RKO cells with or without ACE treatment. **l, m** Flow cytometry analysis showing the cell death of PCBP1-, PCBP2-, and GPX4-knockdown RKO cells. Data are shown as mean ± SEM. n = 3 biological replicates with similar results. All the statistical analysis were performed via one-way ANOVA. **P* < 0.05, ***P* < 0.01, ****P* < 0.001, *****P* < 0.0001; ns not significant.

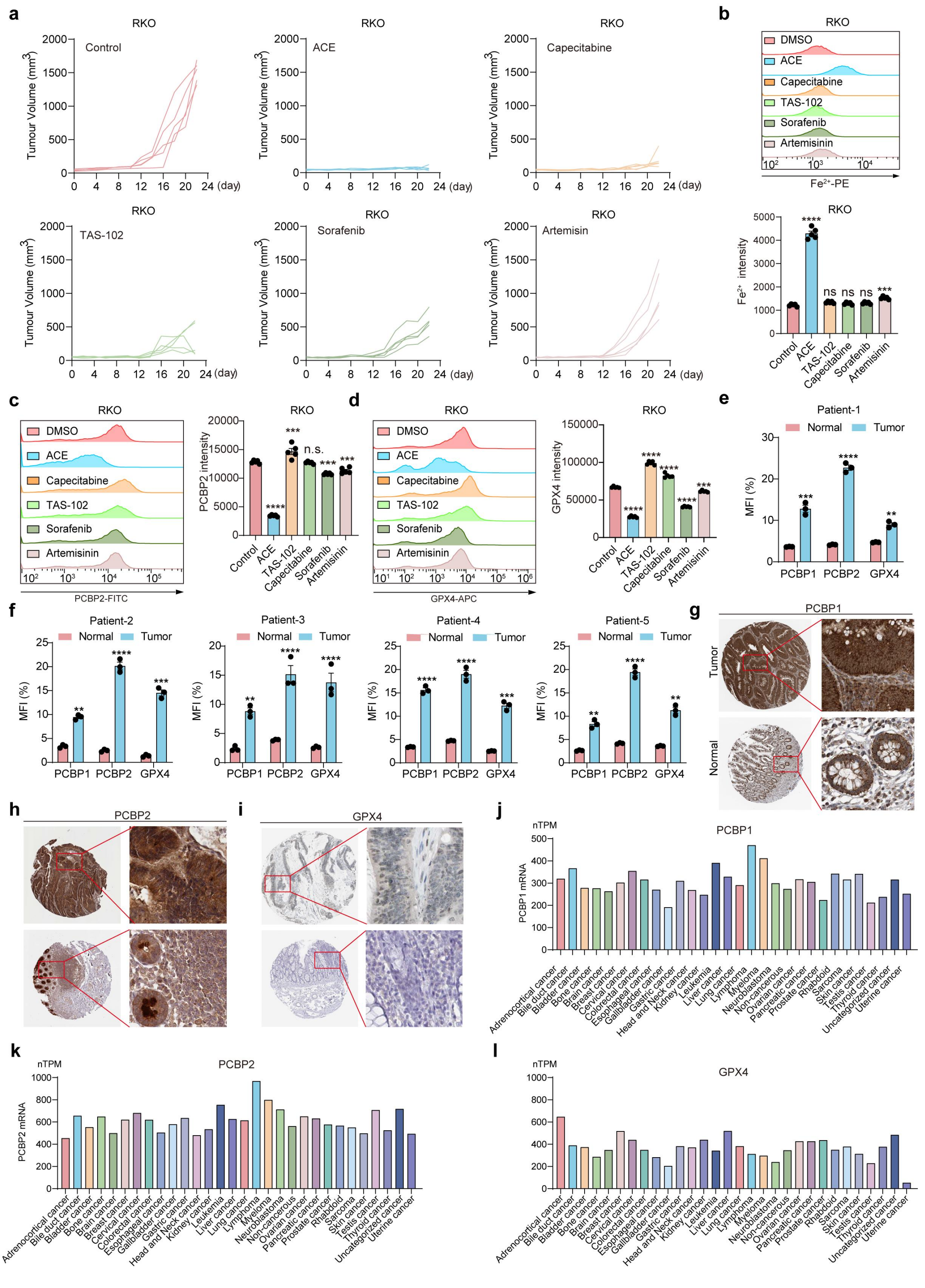


**Supplementary Fig. 15 ACE downregulates PCBP1/2 and GPX4 *in vivo*.**

**a** Tumor volume curves for each mouse in different treatment groups corresponding to **Fig. 8d**. **b-d** Flow cytometry showing levels of $Fe^{2+}$ **(b)**, PCBP2 **(c)**, and GPX4 **(d)** levels in corn oil- or ACE-, capecitabine-, TAS-102-, sorafenib-, and artemisinin-treated RKO mice **e, f**

Quantification of the fluorescence intensity related to **Fig. 8j. g-i** Representative IHC staining for PCBP1 **(g)**, PCBP2 **(h)**, and GPX4 **(i)** in cancer tissues and paracancerous tissues from COAD patients. Scale bars, 200 μm or 50 μm. **J-l** PCBP1 **(j)**, PCBP2 **(k)**, and GPX4 **(l)** mRNA expression in different human cancers based on analysis of the Human Protein Atlas database. The experiments consisted of three biological replicates with similar results. One-way ANOVA was performed in **(b-d).** Student's t-test was performed in **(e, f)**. $^{**}P < 0.01$, $^{***}P < 0.001$, $^{****}P < 0.0001$; ns not significant.

**Table S1: Hematological and biochemical analysis of mice treated orally with ACE (50 mg/kg) for 10 d.**

| Parameters | Control | ACE |
|---|---|---|
| WBC(10/μL) | 581±132.85 | 570.73±98.17 |
| RBC(10^4/μL) | 1228.47±148.9 | 1306.33±69.69 |
| HGB(g/L) | 198.20±21.88 | 211.60±11.70* |
| HCT(%) | 600.13±70.19 | 637.33±35.43 |
| MCV(/fL) | 488.93±5.93 | 487.93±7.00 |
| MCH(/pg) | 161.8±5.91 | 162.00±3.18 |
| MCHC(g/L) | 330.87±9.77 | 332.07±5.60 |
| PLT(10^3/μL) | 1584.87±236.22 | 1674.2±161.59 |
| RDW-SD(/fL) | 231.6±12.68 | 227.33±7.05 |
| RDW-CV(%) | 138.07±9.46 | 133.73±5.08 |
| PDW | 58.93±2.31 | 63.67±5.05** |
| MPV(/fL) | 67.00±1.81 | 69.80±3.82* |
| P-LCR(%) | 40.87±8.98 | 47.80±19.20 |
| PCT()%) | 105.67±14.31 | 116.53±11.61* |
| NEUT#(10/μL) | 21.53±55.99 | 206.53±54.47 |
| LYMPH#(10/μL) | 296.40±78.41 | 294.33±55.38 |
| MONO#(10/μL) | 59.60±29.76 | 57.38±15.64 |
| EO#(10/μL) | 11.47±6.57 | 12.33±7.52 |
| NEUT% | 373.93±79.21 | 360.13±57.80 |

| | | |
|---|---|---|
| LYMPH% | 508.33±80.50 | 517.27±66.76 |
| MONO% | 98.20±35.35 | 100.73±23.43 |
| EO% | 19.53±9.02 | 24.53±9.88 |

The values are presented as mean ± S.D, (15 mice/group) and analyzed by Student's t-test.

**Table S2. Key resources**

| Reagent or Resource | Source | Identifier |
|---|---|---|
| Erastin | APExBio | Cat# B1524 |
| Ferrostatin-1 | MedChemExpress | Cat# HY-100579 |
| Liproxstatin-1 | MedChemExpress | Cat# HY-12726 |
| Deferoxamine | MedChemExpress | Cat# HY-B1625 |
| Chloroquine | MedChemExpress | Cat# HY-17589A |
| Z-VAD-FMK | MedChemExpress | Cat# HY-16658B |
| MG132 | MedChemExpress | Cat# HY-13259 |
| RSL3 | MedChemExpress | Cat# HY-100218A |
| Cycloheximide | MedChemExpress | Cat# HY-12320 |
| Liperfluo | Dojindo | Cat# L248 |
| BODIPY 581/591 C11 | Dojindo | Cat# F374 |
| DAPI | Beyotime Biotechnology | Cat# C1002 |
| PBS | Meilunbio | Cat# MA0015 |
| DMEM | Meilunbio | Cat# MA0212 |
| MEM | Meilunbio | Cat# MA0217 |
| F-12K | Meilunbio | Cat# MA0230 |
| RPMI 1640 | Meilunbio | Cat# MA0548 |
| McCoy's 5A | Meilunbio | Cat# MA0314 |
| DMSO | Beyotime Biotechnology | Cat# ST038 |
| PVDF membranes | Millipore | Cat# ISEQ00010 |
| Lipofectamine 2000 | Thermo Fisher Scientific | Cat# 12566014 |
| Crystal violet | Beyotime Biotechnology | Cat# C0121 |
| FBS | GIBCO | Cat# 25200-056 |
| GSH | Beyotime Biotechnology | Cat# S0073 |
| NAC | MedChemExpress | Cat# HY-B0215 |
| Cell lysis buffer | Beyotime Biotechnology | Cat# P0013F |
| Acevaltrate | Chengdu DesiTe Biological Technology | Cat# DY0294 |
| | | |
| **Antibodies** | | |
| GPX4 | Abcam | Cat# ab125066 |
| GPX4(IP) | Santa Cruze | Cat# sc-166570 |
| HO-1 | Proteintech | Cat# 10701-1-AP |
| FTL | Abmart | Cat# T56955 |

| | | |
|---|---|---|
| NCOA4 | Cell Signaling Technology | Cat# 66849S |
| DMT1 | Cell Signaling Technology | Cat# 15083S |
| PCBP1 | Proteintech | Cat# 14523-1-AP |
| PCBP2 | Proteintech | Cat# 15070-1-AP |
| Snail | Abcam | Cat# ab216347 |
| Slug | Abcam | Cat# ab27568 |
| N-Cadherin | Abcam | Cat# ab245117 |
| Vimentin | Abcam | Cat# ab92547 |
| β-actin | Proteintech | Cat# 20536-1-AP |
| GAPDH | Proteintech | Cat# 60004-1-Ig |
| Tubulin | Proteintech | Cat# 66031-1-Ig |
| | | |
| **Critical Commercial Assays** | | |
| GSH and GSSG Assay Kit | Beyotime Biotechnology | Cat# S0053 |
| GPX Assay Kit | Beyotime Biotechnology | Cat# S0056 |
| MDA Assay Kit | Beyotime Biotechnology | Cat# S0131 |
| BCA Assay Kit | Beyotime Biotechnology | Cat# P0011 |
| Cell Counting Kit-8 | APExBio | Cat# K1018 |
| EdU assay | Beyotime Biotechnology | Cat# ST067 |
| Calcein/PI Cell Viability/Cytotoxicity Assay Kit | Beyotime Biotechnology | Cat# C2015 |
| FITC Annexin V Apoptosis Detection Kit I | BD Biosciences | Cat# AB_2869082 |
| Cell Cycle and Apoptosis Analysis Kit | Beyotime Biotechnology | Cat# C1052 |
| iron Assay | Abcam | Cat# ab83366 |
| | | |
| **Recombinant DNA** | | |
| Plasmid: pcDNA3.1-ub | Guannan Biotechnology | Cat# GN031 |
| Plasmid: pcDNA3.1-GPX4 | Guannan Biotechnology | Cat# GN3866 |
| Plasmid: pcDNA3.1-PCBP1 | Guannan Biotechnology | Cat# GN11886 |
| Plasmid: pcDNA3.1-PCBP2 | Guannan Biotechnology | Cat# GN18266 |
| Plasmid: pcDNA3.1-GFP-GPX4 | Guannan Biotechnology | Cat# GN3867 |
| Plasmid: pcEGFP-PCBP1 | Guannan Biotechnology | Cat# GN11887 |

| | | |
|---|---|---|
| Plasmid: pcEGFP-PCBP2 | Guannan Biotechnology | Cat# GN18267 |
| Plasmid: pcDNA3.1-GFP-GPX4Sec46A | GENEWIZ | Cat# AA62685-4/T1389883 |
| Plasmid: pcEGFP-PCBP1Cys54A | GENEWIZ | Cat# AA62685-1/T1397557 |
| Plasmid: pcEGFP-PCBP1Cys293A | GENEWIZ | Cat# AA62685-2/T1399217 |
| Plasmid: pcEGFP-PCBP2Cys54A | GENEWIZ | Cat# AA62685-3/T1397562 |
| | | |
| **Deposited data** | | |
| RNA-Seq performed with MCF7 cells | ITCM | http://itcm.biotcm.net/download.html |
| Proteins data in TMT6-based proteomics analysis using Acevaltrate in RKO cells | This paper | IPX0012172002 |
| Proteins data in TMT6-based proteomics analysis using Acevaltrate in HCT116 cells | This paper | IPX0012172001 |
| DARTS data of Acevaltrate treated with RKO cells | This paper | IPX0012172003 |
| | | |
| **Oligonucleotides** | **5'-3'** | |
| Human GPX4 qPCR forward primer | AGTCCTGACTACGGCCTCCG | |
| Human GPX4 qPCR reverse primer | GCTCCTGCTTCCCGAACTGG | |
| Human βactin qPCR forward primer | ATTCCTATGTGGGCGACGAG | |
| Human βactin qPCR reverse primer | CCAGATTTTCTCCATGTCGTCC | |
| GPX4-homo-380 forward primer | GGAGUAACGAAGAGAUCAATT | |
| GPX4-homo-380 reverse primer | UUGAUCUCUUCGUUACUCCTT | |
| GPX4-homo-273 forward primer | UUGAUCUCUUCGUUACUCCTT | |
| GPX4-homo-273 reverse primer | GUUGAUCUCUUCGUUACUCCTT | |

| | | |
|---|---|---|
| GPX4-Homo-479 forward primer | GGAAGUGGAUGAAG AUCCATT | |
| GPX4-Homo-479 reverse primer | UGGAUCUUCAUCCA CUUCCTT | |
| PCBP1-homo-288 forward primer | GUGGACUAAAUGUG ACUCUTT | |
| PCBP1-homo-288 reverse primer | AGAGUCACAUUUAG UCCACTT | |
| PCBP1-homo-612 forward primer | GCGGGUGUAAGAUC AAAGATT | |
| PCBP1-homo-612 reverse primer | UCUUUGAUCUUACA CCCGCTT | |
| PCBP1-Homo-1233 forward primer | CUGGUAGGCAGGUU ACUAUTT | |
| PCBP1-Homo-1233 reverse primer | AUAGUAACCUGCCU ACCAGTT | |
| PCBP2-homo-398 forward primer | GGCUACUUAUGCAU GGAAATT | |
| PCBP2-homo-398 reverse primer | UUUCCAUGCAUAAG UAGCCTT | |
| PCBP2-homo-1137 forward primer | CGGAUUCAGUGGCA UUGAATT | |
| PCBP2-homo-1137 reverse primer | UUCAAUGCCACUGA AUCCGTT | |
| PCBP2-Homo-589 forward primer | GAGGACAUAAGCAG CUCUATT | |
| PCBP2-Homo-589 reverse primer | UAGAGCUGCUUAUG UCCUCTT | |
| | | |
| **Software and algorithms** | | |
| Flowjo v10 | Flowjo | https://www.flowjo.com/ |
| Graphpad Prism 9.0 | Graphpad Prism | http://www.graphpad.com/scientific %20software/prism |
| ImageJ | N/A | https://imagej.nih.gov/ij |
